# Metabolic potential of newly isolated bacterial species from the deep subsurface of the Iberian Pyrite Belt

**DOI:** 10.3389/fmicb.2026.1822116

**Published:** 2026-06-09

**Authors:** Adrián Martínez-Bonilla, Guillermo Mateos, Miryam Carrillo-Bautista, David Arranz, Ricardo Amils, Cristina Escudero

**Affiliations:** 1 Centro de Biología Molecular Severo Ochoa (CBM) (CSIC-UAM), Universidad Autónoma de Madrid, Madrid, Spain; 2 Department of Environmental Microbiology, Institute for Sanitary Engineering, Water Quality and Solid Waste Management (ISWA), University of Stuttgart, Stuttgart, Germany; 3 Department of Physiology, Genetics and Microbiology, University of Alicante, Alicante, Spain; 4 Molecular Microbiology Research Laboratory, Department of Technology and Biotechnology of Dairy Products, Dairy Research Institute (IPLA-CSIC), Asturias, Spain; 5 Molecular Microbiology Research Group, Instituto de Investigación Sanitaria del Principado de Asturias (ISPA), Avenida del Hospital Central de Asturias, Oviedo, Spain; 6 Centro de Astrobiología (CAB) (CSIC-INTA), Torrejón de Ardoz, Spain; 7 Geomicrobiology, Department of Geosciences, University of Tübingen, Tübingen, Germany

**Keywords:** bacterial isolation, biogeochemical cycling, deep subsurface microbiology, genome-based analysis, metabolic potential

## Abstract

Río Tinto (Huelva, Spain) is an extreme environment whose origin is a natural underground bioreactor in which the high concentration of metallic sulfides of the Iberian Pyrite Belt are dissolved by microbial activity. As part of a drilling project conducted in the source area of the river, several microorganisms were isolated under strict anaerobic conditions from the deep subsurface of this ecosystem. Here, we report the genomic analysis of these isolates to assess their role in the bioreactor. We sequenced and assembled the genomes of twelve new isolates and studied the potential role of thirteen native microorganisms in the biogeochemical cycles operating in the deep subsurface of the Iberian Pyrite Belt. Our results indicate that seven of the microorganisms belong to new species. Several isolates encode genes associated with metabolic functions relevant for the subsurface bioreactor, including H_2_ production and carbon fixation. In addition, three isolates have the potential to synthesize vitamin B_12_, which could be essential to maintain the microbial community in this ecosystem. Finally, certain genera seem to be highly adapted and widely distributed in the deep subsurface of the Iberian Pyrite Belt.

## 1 Introduction

The deep subsurface is considered an extreme environment characterized by darkness, absence of O_2_ and oligotrophy (Kieft, 2016). In the subsurface, shallow depths might receive photosynthetic products from the surface, while deeper areas can be completely independent from this flux, leading to the so-called Subsurface Lithoautotrophic Microbial Ecosystems (SLiMEs) (Nealson et al., 2005; Stevens and McKinley, 1995). In SLiME systems, H_2_ and CO_2_ are considered the dominant energy and carbon sources, both produced abiotically by geological processes (Stevens and McKinley, 1995). Carbon-fixation pathways generate reduced organic compounds that can support fermentation and other heterotrophic metabolisms (Beaver and Neufeld, 2024). Nevertheless, in environments where organic matter is present, such as those formed through sedimentation processes, fermentative and heterotrophic microorganisms recycle the organic carbon into CO_2_ and H_2_, thereby sustaining the autotrophic microbial community in the subsurface (Parkes et al., 2011; Sanz et al., 2021). Still, other compounds such as reduced nitrogen, sulfur or iron can be used as electron donors and their oxidized forms as electron acceptors (Escudero and Amils, 2023). Due to the deep subsurface’s oligotrophy, coupled metabolisms are especially relevant. Indeed, the end products of one microorganism can be used as electron donors or acceptors by other microorganisms (Beaver and Neufeld, 2024). Therefore, in the deep subsurface the microorganisms establish metabolic interactions, many of which involve the formation of biofilms (Escudero et al., 2018a).

Río Tinto (Huelva, Spain) is an extreme environment whose origin is a bioreactor located in the deep subsurface of the Iberian Pyrite Belt (IPB) (Amils et al., 2023), where the river rises. In this underground bioreactor, the metallic sulfides, mainly pyrite, are dissolved as a result of microbial activity leading to the generation of acidic waters enriched in heavy metals (Gómez-Ortiz et al., 2014).

Two drilling projects have been carried out in the area of Peña de Hierro, in the core of the IPB: Mars Astrobiology Research and Technology Experiment (MARTE) (2003–2006) and Iberian Pyrite Belt Subsurface Life Detection (IPBSL) (2011–2015). During the MARTE project three boreholes were drilled, named BH1, BH4 and BH8 with depths of 59, 166.35 and 166 meters below surface (mbs) respectively. This project had the aim of searching for microbial life in the deep subsurface and to test the technology that could potentially be used on Mars’ subsurface (Fernández-Remolar et al., 2008; Prieto-Ballesteros et al., 2008).

IPBSL was a drilling project aimed at further studying and characterizing the geomicrobiology of the IPB underground bioreactor (Amils et al., 2013). In this project, two boreholes named BH10 and BH11, with depths of 613 and 336 mbs respectively, were drilled (Amils and Fernández-Remolar, 2014). The drilling cores obtained from these boreholes were processed and thoroughly characterized including the assessment of available energy and carbon sources, the identification of coupled metabolic activities and the isolation of several microorganisms from enrichment cultures derived from drilling cores samples (Amils et al., 2023; Leandro et al., 2018).

Previous work sequenced and analyzed the genomes of eight isolates belonging to the most representative microbial genera (*Brevundimonas, Citrobacter, Desulfovibrio, Rhizobium, Rhodoplanes, Shewanella, Stutzerimonas* and *Tessaracoccus*) identified in the deep subsurface of the IPB (Amils et al., 2023; Gallego-Rodríguez et al., 2023; García et al., 2018; Leandro et al., 2017; Mariñán et al., 2019; Martínez et al., 2021, 2024; Mateos et al., 2022; Rodriguez-Robles et al., 2019). These in-depth studies led to the identification of potential metabolic activities and the microbial species candidate for interconnecting the biogeochemical cycles (H, C, N, S and Fe) operating in the deep subsurface of the IPB in the absence of light and O_2_ (Amils et al., 2023). In addition, the analysis of these genomes highlighted the relevant role that vitamin B_12_ plays in this ecosystem (Mateos et al., 2023). Vitamin B_12_ is an essential cofactor for multiple key enzymatic activities (Balabanova et al., 2021). Most analyzed genomes encode vitamin B_12_-dependent enzymes but lack the full biosynthetic pathway. As a result, these microorganisms depend on metabolic interactions with community members capable of *de novo* vitamin B_12_ synthesis.

The aim of this work was to evaluate the metabolic potential of ten additional unstudied isolates from the deep subsurface of the IPB belonging to the genera *Aestuariimicrobium, Cellulomonas, Lelliottia, Microbacterium, Niallia, Nocardioides, Paenibacillus, Pleomorphomonas, Pseudomonas and Propionicimonas*, and to determine their roles in the biogeochemical cycles operating in the underground bioreactor. Moreover, two newly isolated microorganisms of the genera *Rhodoplanes* and *Tessaracoccus* (Martínez-Bonilla, 2025) have been also included to compare them with previously characterized isolates, as these genera are abundant and widely distributed in borehole BH10. Finally, the genome of *Citrobacter telavivensis* T1.2D-1 (Gallego-Rodríguez et al., 2023) was also included in the functional analysis to determine if it contains genes for metabolic pathways not studied before.

## 2 Materials and methods

### 2.1 Isolation and identification of IPB subsurface microorganisms

All the microorganisms used in this project were isolated in strict anaerobic conditions from enrichment cultures established during the IPBSL project using samples from the deep subsurface of the IPB. The methodology employed for the preparation of the enrichment cultures and for the isolation and identification of the microorganisms has already been reported elsewhere (Amils et al., 2023; Leandro, 2018). Briefly, enrichment cultures were inoculated with samples from the deep subsurface of the IPB, specifically from borehole BH10. The targeted metabolisms were nitrate reduction, methanogenesis and nitrate-dependent Fe(II) oxidation (NDFO). Microorganisms were isolated from these enrichment cultures under strict anaerobic conditions using the Hungate roll-tube method (Hungate, 1969). Colonies were screened for unique morphologies and were repeatedly transferred until pure cultures were obtained. After the isolation, the microorganisms were identified by sequencing their 16S rRNA gene using the primers 27F-1492R and were preserved in 20% (v/v) glycerol at −80 °C until further use.

### 2.2 Genome sequencing and assembly

*Aestuariimicrobium* sp. T2.26MG-19.2B, *Lelliottia* sp. T2.23D-8, *Microbacterium* sp. T2.11-28, *Niallia* sp. T2.9-1, *Paenibacillus* sp. T2.5-46A, and *Pseudomonas* sp. T2.1D-1.1 were grown in Tryptic Soy Broth (TSB) (PanReac), whereas *Cellulomonas* sp. T2.22MG-43, *Nocardioides* sp. T2.26MG-1, *Pleomorphomonas* sp. T1.2MG-36, *Propionicimonas* sp. T2.31MG-1, *Rhodoplanes* sp. P11, and *Tessaracoccus* sp. O5.2 were grown in liquid R2A medium (Condalab). All the isolates were incubated in aerobic conditions, at 25 °C and at 130 rpm in a shaker incubator. The DNA was extracted using the cetyltrimethylammonium bromide (CTAB) extraction method (Wilson, 2001). The concentration of the DNA was quantified using a Qubit 2.0 fluorometer (ThermoFisher). The genomes were then sequenced using the Illumina short reads sequencing service of MicrobesNG (Birmingham).

The reads obtained were assembled using the Galaxy platform (The Galaxy Community, 2024). Initially, the assembler SPAdes (Bankevich et al., 2012) (Galaxy Version 3.15.5 + galaxy2) was used. The assembly was carried out with paired-end individual datasets and all the parameters were left as default except for the k-mer values which were set to: “49, 91, 101, 121, 127.” Subsequently, the resulting contigs were further scaffolded using SSPACE (Boetzer et al., 2011) v3.0 for Linux. The library parameters included an expected insert size of 648 bp, a minimum allowed error of 0.5 and FR orientation. The extension option was selected.

The draft genomes were compared with the closest phylogenetically related microorganism with an available sequenced genome based on their 16S rRNA gene sequence similarity. The reference genomes used along with their accession number from GenBank were: *Aestuariimicrobium kwangyangense* DSM 21549 (ATXE01000001.1), *Cellulomonas fimi* ATCC 484 (CP002666.1), *Lelliottia amnigena* JA266 (CP079896.1), *Microbacterium saccharophilum* K-1 (VRSX01000001.1), *Niallia circulans* NBRC 13626 (NZ_BCVE01000001.1), *Nocardioides pyridinolyticus* JCM 10369 (BBGV01000001.1), *Paenibacillus odorifer* DSM 15391 (NZ_CP009428.1), *Pleomorphomonas oryzae* DSM 16300 (AUHB01000001.1), *Propionicimonas paludicola* DSM 1597 (PDJC01000001.1), *Pseudomonas aeruginosa* JCM 5962 (GCA_000615485.1), *Rhodoplanes serenus* DSM 18633 (GCA_009720755.1), and *Tessaracoccus lapidicaptus* IPBSL-7 (GCF_001693815.1).

### 2.3 Genomic indexes

Genomic robust indexes, Average Amino acid Identity (AAI) (Konstantinidis and Tiedje, 2005), Average Nucleotide Identity (ANI) (Yoon et al., 2017) and digital DNA–DNA Hybridization (dDDH) (Meier-Kolthoff et al., 2022), were calculated for a more precise taxonomic classification. AAI was calculated using the tool EzAAI, ANI was calculated with the “ANI Calculator” tool from EzBioCloud and dDDH was calculated with the “Genome-to-Genome Distance Calculator 3.0” (GGDC) tool from the DSMZ. The genomes of the isolates from the deep subsurface of the IPB were first compared to a reference genome of the closest species based on their 16S rRNA sequence.

In the cases where the isolates belonged to a species different from the reference used, the microorganisms were compared with up to four other representative species from the same genus. The species chosen were those with highest 16S rRNA sequence identity with the studied isolates.

### 2.4 Functional analysis

The draft genomes were annotated using the program Prokka (Seemann, 2014) (Galaxy Version 1.14.6 + galaxy1) with all parameters set as default except for the “compliant” argument. The annotations were studied to identify genes related to nitrogen, sulfur, carbon, hydrogen and iron cycles, as well as genes involved in vitamin B_12_ biosynthesis or acquisition. In those cases where a gene was not found in the annotation, its presence in the genome was double-checked using protein BLAST (blastp) and the amino acid sequence of the protein retrieved from UniProt (The UniProt Consortium, 2018). For a result to be considered positive, it required a percentage of identity higher than 35%, a coverage higher than 50% and an E-value below 0.001 (Pearson, 2013).

The annotation of *C. telavivensis* T1.2D-1 (accession number GCA_950098555.1 in GenBank), a microorganism isolated from the deep subsurface of the IPB whose genome has been previously sequenced and annotated (Gallego-Rodríguez et al., 2023), was also included in this functional analysis, as some of the activities studied in this study were not previously analyzed in this genome.

### 2.5 Genomic similarity between the isolates of the genera *Tessaracoccus* and *Rhodoplanes*

The genomes of *Rhodoplanes serenus* P11 and *Tessaracoccus lapidicaptus* O5.2 were compared with *Rhodoplanes serenus* T2.26MG-98 and *Tessaracoccus lapidicaptus* T2.5-30, two microorganisms of the same species previously isolated from the deep subsurface of the IPB and characterized (Amils et al., 2023). dDDH was calculated as previously described and ANI and AAI were determined using the programs ani.rb and aai.rb from the enveomics collection (Rodríguez-R and Konstantinidis, 2016).

## 3 Results

### 3.1 Genome sequencing, assembly and annotation

The aim of this work was to study the metabolic potential of twelve microorganisms from the deep subsurface of the IPB, ten of which had been previously isolated (Amils et al., 2023; Leandro et al., 2018) but not yet characterized: *Aestuariimicrobium* sp. T2.26MG-19.2B, *Cellulomonas* sp. T2.22MG-43, *Lelliottia* sp. T2.23D-8, *Microbacterium* sp. T2.11-28, *Niallia* sp. T2.9-1, *Nocardioides* sp. T2.26MG-1, *Paenibacillus* sp. T2.5-46A, *Pleomorphomonas* sp. T1.2MG-36, *Propionicimonas* sp. T2.31MG-1, and *Pseudomonas* sp. T2.1D-1.1; and two microorganisms newly isolated from active enrichment cultures: *Rhodoplanes* sp. P11 and *Tessaracoccus* sp. O5.2 (Martínez-Bonilla, 2025). To address this aim, the genomes of all twelve microorganisms were sequenced, assembled and annotated (Table 1) to assess their metabolic potential.

**Table 1:** Parameters obtained from the sequencing and assembly of the strains isolated from the deep subsurface of the IPB.

Isolate	Number of reads	Mean coverage	Median insert size	Number of contigs	Size (bp)	N50	GC (%)	Number of proteins
*Aestuariimicrobium* sp. T2.26MG-19.2B	363,858	55.11	659	8	3,021,769	2,202,518	68.42	2,675
*Cellulomonas* sp. T2.22MG-43	828,420	102.67	710	5	3,747,951	934,638	73.19	3,420
*Lelliottia* sp. T2.26D-8	1,047,983	109.35	630	41	4,570,637	445,083	52.8	4,317
*Microbacterium* sp. T2.11-28	327,043	43.79	638	33	3,401,241	325,394	71.22	3,199
*Niallia* sp. T2.9-1	1,457,320	149.40	630	162	4,628,314	76,228	35.36	4,555
*Nocardioides* sp. T2.26MG-1	282,167	24.99	611	37	5,139,621	428,377	72.04	5,008
*Paenibacillus* sp. T2.5-46A	1,542,203	108.27	648	62	6,933,836	551,861	43.56	6,099
*Pleomorphomonas* sp. T1.2MG-36	979,865	89.54	603	54	5,198,610	952,541	64.73	4,698
*Propionicimonas* sp. T2.31MG-1	1,123,512	132.72	730	31	3,946,155	534,157	71.36	3,562
*Pseudomonas* sp. T2.1D-1.1	2,636,746	198.89	564	29	6,342,513	827,838	66.49	5,838
*Rhodoplanes* sp. P11	1,437,790	120.09	628	26	5,591,448	580,082	70.25	5,052
*Tessaracoccus* sp. O5.2	633,807	100	664	60	2,995,752	141,150	70.36	2,846

The parameters indicated for each genome are: number of reads obtained from the sequencing, mean coverage of the sequencing, median insert size, numbers of contigs obtained after the assembly, length of the genome, N50 of the genome, percentage of GC of the genome and number of proteins encoded in the genome.

The draft genomes were compared with the closest known microorganisms based on 16S rRNA gene sequence similarity. Genome size, GC content and predicted protein number of the genomes were compared with those of the reference genomes (Supplementary Table S1). All the isolates showed a GC content similar to their respective references. However, some differences were observed in the genome size and in the number of predicted proteins. For instance, *Cellulomonas* sp. T2.22MG-43 and *Niallia* sp. T2.9-1 had smaller genomes and less predicted proteins, while *Microbacterium* sp. T2.11-28, *Nocardioides* sp. T2.26MG-1 and *Propionicimonas* sp. T2.31MG-1 had larger genomes and encoded more proteins than their corresponding reference genomes. Although there is no consistent pattern across all isolates, these differences suggest that some of the isolates may be more distantly related to their references than indicated by 16S rRNA gene sequence identity alone.

### 3.2 Genomic indexes

Since the 16S rRNA gene sequence has limited resolution for distinguishing closely related species (Kim et al., 2014), three genomic indexes (AAI, ANI and dDDH) were calculated to obtain a more robust taxonomic classification of the isolates (Table 2). Each of the isolates was compared to the closest strain inferred from their 16S rRNA sequence.

**Table 2:** Genomic indexes obtained from the comparison of the isolates with their closest strains.

Isolate	Closest strain	16S	AAI	ANI	dDDH
*Aestuariimicrobium* sp. T2.26MG-19.2B	*Aestuariimicrobium kwangyangense* DSM 21549	99.93	98.77*	98.36*	85.60*
*Cellulomonas* sp. T2.22MG-43	*Cellulomonas fimi* ATCC 484	97.65	70.61	76.40	20.50
*Lelliottia* sp. T2.26D-8	*Lelliottia amnigena* JA266	100	98.77*	98.40*	86.40*
*Microbacterium* sp. T2.11-28	*Microbacterium saccharophilum* K-1	99.42	77.42	79.32	22.00
*Niallia* sp. T2.9-1	*Niallia circulans* NBRC 13626	98.98	87.32	82.76	26.40
*Nocardioides* sp. T2.26MG-1	*Nocardioides pyridinolyticus* JCM 10369	97.9	79.04	83.02	29.10
*Paenibacillus* sp. T2.5-46A	*Paenibacillus odorifer* DSM 15391	98.84	90.4	86.00	30.50
*Pleomorphomonas* sp. T1.2MG-36	*Pleomorphomonas oryzae* DSM 16300	99.7	87.83	84.22	27.60
*Propionicimonas* sp. T2.31MG-1	*Propionicimonas paludicola* DSM 1597	98.53	72.75	76.96	20.70
*Pseudomonas* sp. T2.1D-1.1	*Pseudomonas aeruginosa* JCM 5962	100	99.46*	99.37*	95.00*
*Rhodoplanes* sp. P11	*Rhodoplanes serenus* DSM 18633	99.6	97.72*	97.12*	85.10*
*Tessaracoccus* sp. O5.2	*Tessaracoccus lapidicaptus* IPBSL-7	99.93	97.86*	97.48*	79.20*

The table shows the 16S rRNA gene sequence identity and the corresponding AAI, ANI and dDDH values. Values exceeding the species threshold are marked with an asterisk.

Each of the genomic indexes has an established species-level threshold to discriminate if two isolates belong to the same species: 95% for AAI (Luo et al., 2014), 95–96% for ANI (Yoon et al., 2017) and 70% for dDDH (Meier-Kolthoff et al., 2013). In our dataset the values of the three indexes were higher than their respective thresholds for *Aestuariimicrobium* sp. T2.26MG-19.2B, *Lelliottia* sp. T2.23D-8, *Pseudomonas* sp. T2.1D-1.1, *Rhodoplanes* sp. P11 and *Tessaracoccus* sp. O5.2, indicating that these isolates belong to previously described species. Accordingly, the following isolates can be assigned to known taxa: *Aestuariimicrobium kwangyangense* T2.26MG-19.2B, *Lelliottia amnigena* T2.23D-8, *Pseudomonas aeruginosa* T2.1D-1.1, *Rhodoplanes serenus* P11 and *Tessaracoccus lapidicaptus* O5.2.

In contrast, *Cellulomonas* sp. T2.22MG-43, *Microbacterium* sp. T2.11-28, *Niallia* sp. T2.9-1, *Nocardioides* sp. T2.26MG-1, *Paenibacillus* sp. T2.5-46A, *Pleomorphomonas* sp. T1.2MG-36 and *Propionicimonas* sp. T2.31MG-1 showed values below the species-level thresholds, supporting their classification as distinct species. To corroborate these results, the genomic indexes were recalculated by comparing each isolate with the genomes of the four next closest species within the same genus based on the 16S rRNA sequence similarity (Supplementary Table S2). In all cases, the AAI, ANI and dDDH values were below the species threshold, further supporting the hypothesis that these isolates belong to new species within their respective genera. For the isolate assigned to the genus *Propionicimonas,* this additional analysis could not be conducted since the only other described species in this genus, *Propionicimonas ferrireducens* (Zhou et al., 2018), lacks a publicly available genome. Thus, based on the comparison with the genome of *P. paludicola,* this isolate likewise represents a new species.

### 3.3 Metabolic potential of the isolates in subsurface biogeochemical cycles

**Figure 1:**
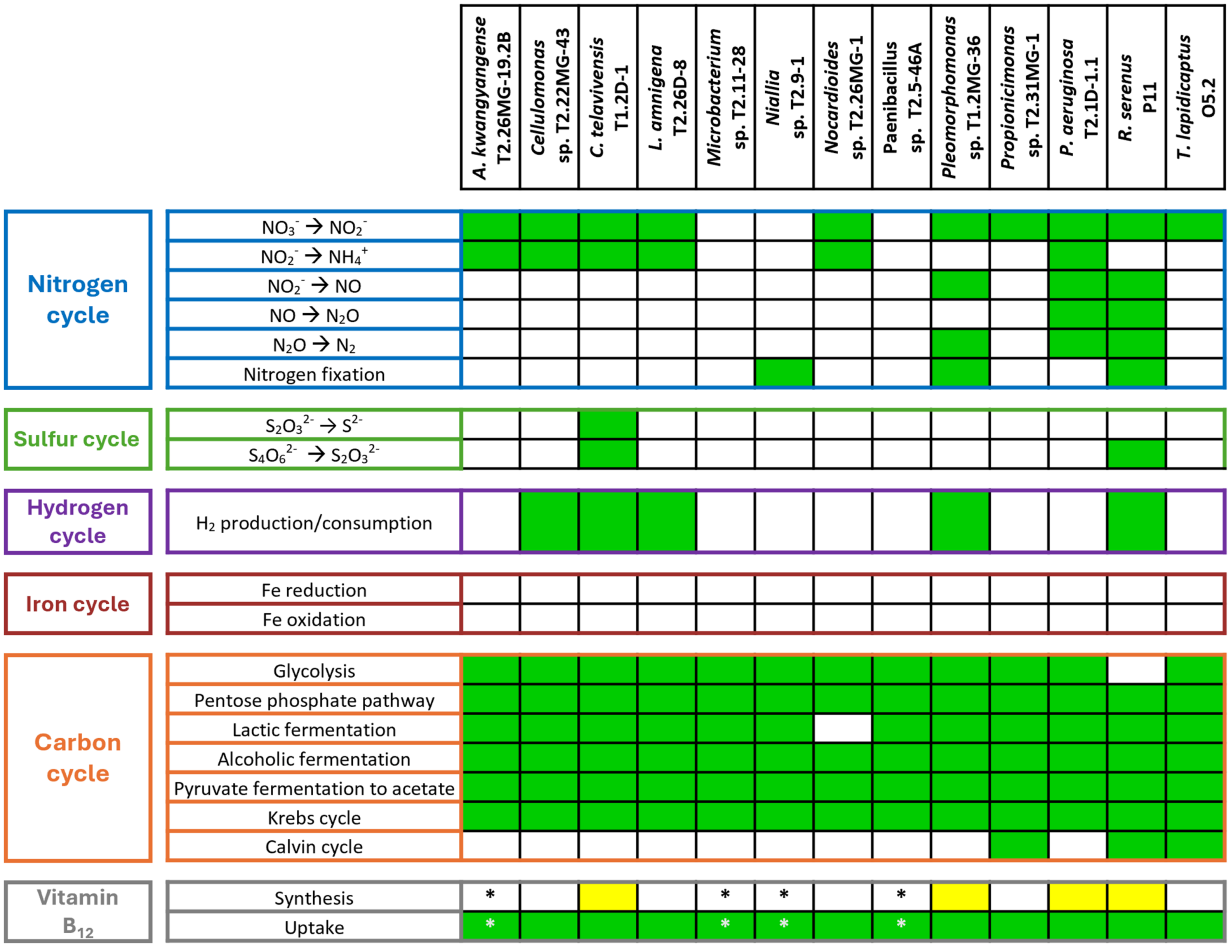
Metabolic pathways identified in the studied genomes. Pathways associated with the nitrogen (blue), sulfur (green), hydrogen (purple), and carbon (orange) cycles, as well as vitamin B_12_-related pathways (grey), are shown. Green squares indicate that all genes required for a given pathway were identified in the genome; yellow squares indicate that most genes were present; and white squares indicate that most genes were absent. An asterisk (*) indicates that the vitamin B_12_-related pathways were previously reported by Mateos et al. (2023). No genes associated with iron oxidation or reduction were identified in any of the analyzed genomes; therefore, iron-related pathways are not included in the figure.

The genome annotations were examined to identify genes related to the nitrogen (reduction of nitrate, nitrite, nitric oxide, and nitrous oxide; nitrogen fixation and dissimilatory nitrate reduction to ammonium), sulfur (reduction of sulfate, sulfite, thiosulfate, tetrathionate and polysulfide; and oxidation of sulfide, thiosulfate and sulfite), iron (iron oxidation and reduction), hydrogen (hydrogen production and consumption) and carbon (catabolic pathways such as glycolysis, pentose phosphate pathway, lactic fermentation, alcoholic fermentation, propionate formation, pyruvate fermentation to acetate and Krebs cycle; and anabolic pathways such as Calvin cycle and Wood-Ljungdahl pathway) cycles, as well as genes related to vitamin B_12_ (biosynthesis and uptake) (Supplementary Figure S1). This analysis provided insights into the potential contributions of the isolates to the biogeochemical cycles previously described for the underground bioreactor of the IPB (Amils et al., 2023). The results are summarized in Figure 1. The genomic analysis of an additional IPB subsurface isolate, *C*. *telavivensis* T1.2D-1, has been included due to the lack of information on the metabolic capabilities of this isolate.

Among the analyzed pathways, nitrogen-related genes were the most widely distributed across the isolates. Ten out of the thirteen isolates (except for *Microbacterium* sp. T2.11-28*, Niallia* sp. T2.9-1 and *Paenibacillus* sp. T2.5-46A) harbor the complete set of genes required for NO_3_^−^ reduction (*narGHI* and/or *napAB*, which encode for the nitrate reductase and the periplasmic nitrate reductase respectively) (Figure 1).

Three isolates (*Pleomorphomonas* sp. T1.2MG-36*, P. aeruginosa* T2.1D-1.1 and *R. serenus* P11) contain the full set of genes required for denitrification pathway (*nirK* or *nirS*, *norBC* and *nosZ*, encoding copper- or cytochrome cd1-containing nitrite reductase, nitric oxide reductase and nitrous oxide reductase, respectively) (Figure 1). Thus, they can potentially use a broader range of nitrogen species as electron acceptors. Additionally, six isolates (*A. kwangyangense* T2.26MG-19.2B, *Cellulomonas* sp. T2.22MG-43, *C. telavivensis* T1.2D-1, *L. amnigena* T2.26D-8, *Nocardioides* sp. T2.26MG-1, and *P. aeruginosa* T2.1D-1.1) possess genes for Dissimilatory Nitrate Reduction to Ammonium (DNRA) (*nirBD*, encoding nitrite reductase), indicating that they may contribute to NH_4_^+^ production.

Three of the isolates (*Niallia* sp. T2.9-1*, Pleomorphomonas* sp. T1.2MG-36 and *R. serenus* P11) have the necessary genes required for nitrogen fixation (*nifKDH*, encoding nitrogenase). This metabolism is also significant as it produces not only NH_4_^+^ which could be used by ANAMMOX microorganisms (Amils et al., 2023) but also H_2_ (Bothe et al., 2010), a key electron donor in the deep subsurface, as discussed below.

In contrast to nitrogen metabolism, sulfur-related pathways were less frequently detected. The genome of *C. telavivensis* T1.2D-1 contains genes for utilizing thiosulfate (*phsABC*, encoding thiosulfate reductase) and tetrathionate (*ttrABC*, encoding tetrathionate reductase) as electron acceptors, the latter also being present in the genome of *R. serenus* P11. These findings suggest that these microorganisms might contribute to the S cycle in this ecosystem.

Hydrogen metabolism represented another potentially relevant energy-conserving strategy identified in several isolates. Five of the isolates (*Cellulomonas* sp. T2.22MG-43*, C. telavivensis* T1.2D-1*, L. amnigena* T2.26D-8*, Pleomorphomonas* sp. T1.2MG-36 and *R. serenus* P11) encode hydrogenases in their genomes (Figure 1). Specifically, they contain hydrogenases homologous to those of *Escherichia coli*: Hyd-1 (*hya* genes), Hyd-2 (*hyb* genes), Hyd-3 (*hyc* genes), and/or Hyd-4 (*hyf* genes) (Vardar-Schara et al., 2008). These enzymes have been reported to be reversible, being able to both produce and oxidize H_2_, suggesting that these microorganisms may switch between H_2_ consumption or production depending on environmental conditions, such as pH and carbon source availability (Mirzoyan et al., 2017). In addition, four isolates (*Cellulomonas* sp. T2.22MG-43*, C. telavivensis* T1.2D-1*, L. amnigena* T2.26D-8*, Pleomorphomonas* sp. T1.2MG-36) encode *hyd* and *hnd* genes, which have also been associated with reversible H_2_ metabolism (Kpebe et al., 2023; McGlynn et al., 2007).

Regarding iron metabolism, none of the analyzed isolates encode known genes related to enzymatic (direct) Fe(II) oxidation or reduction. Consequently, no canonical iron redox pathways are displayed in Figure 1.

Genes required for the utilization of diverse carbon sources as electron donors have been identified in all the genomes of the isolates, including both respiration and fermentation (Figure 1). Notably, three isolates, *Propionicimonas* sp. T2.31MG-1*, R. serenus* P11 and *T. lapidicaptus* O5.2, contain genes encoding the RuBisCO (*ccbLS*; Ribulose-1,5-Bisphosphate Carboxylase/Oxygenase) (Figure 1) along with other genes from the Calvin cycle.

In addition to central biogeochemical pathways, the potential for vitamin B12 biosynthesis was also evaluated. Four of the isolates (*C. telavivensis* T1.2D-1*, Pleomorphomonas* sp. T1.2MG-36, *P. aeruginosa* T2.1D-1.1 and *R. serenus* P11) (Figure 1) contain most of the genes required for the synthesis of vitamin B_12_, although they do not encode for the complete pathway. However, previous studies have shown that some vitamin B_12_ synthesizers lack some genes from the pathway, while sharing a conserved core set of genes (Shelton et al., 2019). In the case of these four isolates, this core gene set is present in their genomes (Supplementary Figure S1). Therefore, these four microorganisms are likely capable of synthesizing vitamin B_12_.

### 3.4 Genomic similarity

*R. serenus* P11 and *T. lapidicaptus* O5.2 were compared with closely related strains of the same species previously obtained from different depths of borehole BH10: *R. serenus* T2.26MG-98 and *T. lapidicaptus* T2.5-30 (Table 3). In both cases, the isolates showed high value for all the genomic indexes.

**Table 3:** Similarity of the isolates of *R. serenus* and *T. lapidicaptus* from the deep subsurface of the IPB.

Isolate	Size (bp)	GC (%)	Number of proteins	16S	AAI	ANI	dDDH
*R. serenus* P11	5,591,448	70.25	5,052	99.93	99.97	99.96	100
*R. serenus* T2.26MG-98	5,589,602	70.24	4,994				
*T. lapidicaptus* O5.2	2,995,752	70.36	2,846	99.93	97.69	97.48	79.2
*T. lapidicaptus* T2.5-30	3,212,699	70.4	3,005				

The table shows the parameters obtained by the comparison of the two microorganisms from the species *R. serenus* and *T. lapidicaptus* sequenced in this project (P11 and O5.2) and the two isolates previously characterized (T2.26MG-98 and T2.5-30). The table includes the size (in bp), the content in GC in percentage, the number of proteins coded in each of the genomes, the percentage of sequence identity between the 16S rRNA sequence of the isolates from the same genus and the taxonomic indexes from the comparison of the same pairs of isolates.

## 4 Discussion

### 4.1 Role of the isolates in the biogeochemical cycles of the IPB deep subsurface

#### 4.1.1 Widespread nitrate reduction and isolates capable of denitrification and nitrogen fixation

**Figure 2:**
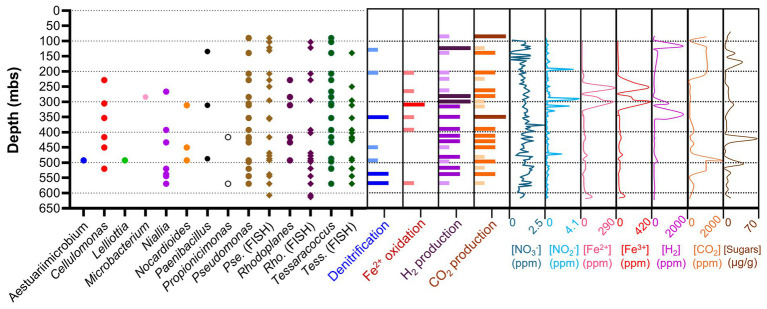
Distribution of microorganisms and metabolic activities across different depths in borehole BH10. Circles indicate the depths (mbs) from which the microorganisms analyzed in this study were isolated, together with additional detection depths previously identified by DNA sequencing and immunological techniques during the IPBSL project (Amils et al., 2023). Diamonds indicate the distribution of *Pseudomonas* (*Pse.*), *Rhodoplanes* (*Rho.*), and *Tessaracoccus* (*Tess.*) additionally assessed by CARD-FISH (Amils et al., 2023). Bars represent the depths at which denitrification (blue), Fe(II) oxidation (red), H_2_ production (purple), and CO_2_ production (orange) were previously detected in anaerobic enrichment cultures prepared from BH10 samples during the IPBSL project (Amils et al., 2023). Bar length and color intensity are proportional to the detected metabolic activity. Concentration profiles of compounds associated with the detected biogeochemical cycles (NO_3_^−^, NO_2_^−^, Fe^2+^, Fe^3+^, H_2_, CO_2_, and carbohydrates) are also shown. Concentrations are expressed in ppm, except for carbohydrates, which are expressed in μg g^−1^ rock. *C. telavivensis* T1.2D-1 and *Pleomorphomonas* sp. T1.2MG-36 are not included because they were isolated from borehole BH11.

Eleven out of the thirteen isolates (with exception of *Microbacterium* sp. T2.11-28 and *Paenibacillus* sp. T2.5-46A) possess genes associated with the N cycle (Figure 1). This widespread genetic potential highlight nitrogen transformations as one of the dominant metabolic features of the IPB deep subsurface. Several microorganisms previously isolated from this environment can actively participate in different nitrogen transformations (Amils et al., 2023). Nitrate reduction has also been described in related species such as *A. kwangyangense* (Jung et al., 2007), *Cellulomonas fimi* (Stackebrandt and Kandler, 1979), *L. amnigena* (Thakur and Gauba, 2023), *Nocardioides pyridinolyticus* (Yoon et al., 1997), and *T. lapidicaptus* (Puente-Sanchez et al., 2014), and denitrification has also been demonstrated in *Pseudomonas aeruginosa* (Arat et al., 2015) and *R. serenus* (Okamura et al., 2009), further supporting the genomic-based predictions presented here. Furthermore, NO_3_^−^ was detected throughout borehole BH10 and other N species, such as NO_2_^−^ and NH_4_^+^, have also been detected at several depths, indicating the presence of an active N cycle in this ecosystem (Figure 2). In addition, denitrifying activity has been observed in enrichment cultures from several depths (Amils et al., 2023). Taken together, geochemical data and genomic evidence suggest that NO_3_^−^ likely represents a major electron acceptor sustaining microbial respiration at depth. NH_4_^+^, in turn, could be used as electron donor by other microorganisms, such as ANAMMOX, which have also been detected in the system (Amils et al., 2023).

Although both NO_3_^−^ and NH_4_^+^ are consistently detected in the IPB subsurface, their origin remains unclear (Amils et al., 2023), raising questions about how nitrogen availability is sustained at depth. In oligotrophic and energy-limited subsurface environments, nitrogen acquisition strategies are expected to be tightly constrained by both substrate availability and energetic costs. That is to say, under IPB subsurface conditions, microorganisms are likely to assimilate nitrogen primarily as NH_4_^+^ from the extracellular environment or by DNRA using NO_3_^−^ (Van Heeswijk et al., 2013) without necessarily relying on the energetically costly process of N_2_ fixation. However, at depths where NH_4_^+^ availability is limited, the nitrogenase activity encoded by some isolates may provide a selective advantage, allowing them to colonize and thrive in nitrogen-depleted microniches.

Nevertheless, it might be worth considering that nitrogen-fixing microorganisms could also represent a primary input of bioavailable nitrogen into the system, potentially contributing to the elevated NH_4_^+^ and overall nitrogen concentrations observed in the IPB subsurface, or at least preventing nitrogen limitation in this ecosystem. Thus, nitrogen fixation may function not only as a survival strategy under local scarcity, but also as a community-level mechanism buffering nitrogen availability. Although nitrogen fixation is energetically demanding, the presence of substantial amounts of organic matter in the IPB subsurface, together with the documented biological production of H_2_ from this organic matter, suggests that energy availability may be less limiting than generally assumed for deep subsurface environments, potentially providing the energetic support required to sustain nitrogen fixation. This potential role is further supported by previous reports of nitrogen-fixing microorganisms in the IPB deep subsurface, including *Desulfosporosinus meridei* DEEP and two cyanobacteria (Amils et al., 2023), as well as by evidence of nitrogen fixation in *Niallia circulans* (Hafez et al., 2025), *Pleomorphomonas oryzae* (Xie and Yokota, 2005) and *R. serenus* (Okamura et al., 2009).

#### 4.1.2 Thiosulfate and tetrathionate as electron donors

During the IPBSL project, SO_4_^2−^ was the only sulfur species analyzed along the borehole. Thus, the presence, distribution and concentration of other sulfur intermediates such as S_2_O_3_^2−^ or S_4_O_6_^2−^ in the IPB subsurface remain unknown. This analytical limitation constrains our ability to fully assess the quantitative relevance of intermediate sulfur species *in situ*. Nevertheless, previous studies have reported the presence of microorganisms capable of participating in the sulfur cycle in this environment, including sulfate-reducing and sulfur-oxidizing microorganisms (Amils et al., 2023).

In line with these previous observations, the genomic data obtained in this study indicate that at least some of the analyzed isolates harbor genes associated with sulfur transformations, including S_2_O_3_^2−^ and S_4_O_6_^2−^ reduction. Moreover, tetrathionate reduction has already been experimentally observed in *Citrobacter* sp. T1.2D-12 (Gallego-Rodríguez et al., 2023), supporting the functional relevance of sulfur-based metabolisms inferred from genomic data in this study. Although sulfur-related genes were less widespread than those involved in nitrogen metabolism, their presence suggests that sulfur cycling may contribute to redox balance in specific microniches within the subsurface.

Given the complex mineralogy of the IPB and the abundance of sulfide-bearing minerals, even low concentrations of reactive sulfur intermediates could support localized sulfur-based metabolisms. Therefore, while nitrogen transformations appear to dominate the metabolic landscape, sulfur cycling may represent an additional, spatially heterogeneous component of the underground bioreactor.

#### 4.1.3 Hydrogen production in the deep subsurface of the IPB

H_2_ is considered one of the most important electron donors in the deep subsurface (Beaver and Neufeld, 2024), particularly in SLiMEs. In the IPB subsurface, H_2_ has been detected occluded within rock samples, in enrichment cultures, and biological production has been demonstrated upon incubation of deep subsurface rock samples with mineral medium (Figure 2). Although the origin of H_2_ in the deep subsurface is often attributed to abiotic processes (Nealson et al., 2005), these findings demonstrated that it can also be produced biologically in the IPB subsurface (Sanz et al., 2021).

Both biotic and abiotic H_2_ can support methanogens, acetogens and other hydrogenotrophic microorganisms. These processes, in turn, produce reduced organic carbon which can be used by heterotrophic microorganisms capable of fermentation or other catabolic pathways coupled to electron acceptors such as NO_3_^−^, SO_4_^2−^ or Fe^3+^.

The genomic data obtained in this study further support the ecological relevance of hydrogen cycling in the IPB subsurface. *Cellulomonas* sp. T2.22MG-43*, C. telavivensis* T1.2D-1*, L. amnigena* T2.26D-8*, Pleomorphomonas* sp. T1.2MG-36 and *R. serenus* P11 could potentially produce and consume H_2_ in this ecosystem, in agreement with previous studies demonstrating hydrogen production in *C. fimi* (Hitit et al., 2017), *L. amnigena* (Halvorsen, 2024), *P. oryzae* (Esquivel-Elizondo et al., 2018) and *R. serenus* (Okamura et al., 2009). The presence of reversible hydrogenases suggests metabolic flexibility, allowing these microorganisms to switch between H_2_ production and consumption depending on local redox conditions, a capability that has also been experimentally confirmed in *Citrobacter* sp. T1.2D-12 (Gallego-Rodríguez et al., 2023).

#### 4.1.4 Metabolic potential for NDFO and chemodenitrification

Although none of the isolates contain genes related to the iron cycle, iron redox transformations may still occur indirectly through nitrogen-driven processes. Microorganisms can potentially carry out NDFO through a chemodenitrification mechanism (Bryce et al., 2018), a metabolism previously reported in isolates from the IPB (Amils et al., 2023). In this process, Fe(II) oxidation would not be enzymatically catalyzed, but instead mediated by the reactive nitrogen species generated during the denitrification (nitrite and nitric oxide) (Bryce et al., 2018). In fact, some authors have suggested that all nitrate reducers are capable of NDFO through the reactive nitrogen intermediates generated during anaerobic respiration (Carlson et al., 2013).

Fe(II) was also detected throughout most of the depths of borehole BH10 (Figure 2). Given the widespread genetic potential for NO_3_^−^ reduction identified in this study, NDFO emerges as a plausible link between the nitrogen and iron cycles in the IPB subsurface. Ten isolates have all the genes necessary to reduce NO_3_^−^, making them plausible candidates for NDFO in this ecosystem. In the case of *C*. *telavivensis* T1.2D-1 this capability has already been demonstrated experimentally (Gallego-Rodríguez et al., 2023). The remaining nine isolates with identical genetic potential (*A. kwangyangense* T2.26MG-19.2B, *Cellulomonas* sp. T2.22MG-43*, L. amnigena* T2.26D-8, *Nocardioides* sp. T2.26MG-1*, Pleomorphomonas* sp. T1.2MG-36*, Propionicimonas* sp. T2.31MG-*, P. aeruginosa* T2.1D-1.1 and *R. serenus* P11 and *T. lapidicaptus* O5.2) (Figure 1) may therefore also be capable of carrying out NDFO, although experimental validation is required, which is consistent with experimental evidence for NDFO in members of the genera *Cellulomonas* (Peng et al., 2019), *Nocardioides* (Nordhoff et al., 2017), *Pseudomonas* (Feng Su et al., 2015; Zhang et al., 2015) and *T. lapidicaptus* (Amils et al., 2023).

NDFO metabolism is considered key in this ecosystem because it may contribute to the high concentrations of Fe(III) characteristic of Río Tinto. The leading hypothesis attributes the extreme conditions of the river, i.e., low pH and high Fe(III) levels, to the dissolution of metal sulfides, mainly pyrite, in the deep subsurface (Gómez-Ortiz et al., 2014). Under IPB subsurface conditions, the Fe(III) generated during NDFO may subsequently promote the oxidation of pyrite, thereby amplifying iron mobilization in the system (Bosch and Meckenstock, 2012; Moses et al., 1987). Thus, even in the absence of canonical iron-oxidizing genes, nitrate-reducing microorganisms may indirectly drive iron cycling and contribute to the long-term geochemical evolution of the IPB system.

#### 4.1.5 Fermentation and carbon fixation

Studies of the deep subsurface of the IPB have reported significant concentrations of organic carbon compounds, including carbohydrates, proteins, and organic acids such as acetate, formate, propionate, and oxalate (Figure 2). These substrates can support microbial activity through anaerobic respiration or fermentation. All isolates have genes to perform fermentative pathways, such as lactic and alcoholic fermentation, suggesting that they could thrive in this ecosystem even in the absence of suitable electron acceptors. In fact, *Microbacterium* sp. T2.11-28*, Niallia* sp. T2.9-1 and *Paenibacillus* sp. T2.5-46A lack genes associated with known anaerobic respiratory pathways, indicating they might rely primarily on fermentation for energy production.

At the same time, the presence of CO_2_ in the deep subsurface (Amils et al., 2023) opens the possibility for autotrophic or mixotrophic strategies. Some of the analyzed isolates encode genes associated with carbon fixation pathways, suggesting that inorganic carbon assimilation may complement heterotrophic growth and potentially supply organic carbon to the surrounding microbial community.

Carbon cycling in the IPB subsurface therefore likely involves a dynamic balance between organic matter degradation and inorganic carbon fixation, coupled to the nitrogen, sulfur and iron redox processes discussed above. All microorganisms isolated in this project are facultative anaerobes and therefore retain the capacity to perform aerobic respiration when oxygen becomes available. Although oxygen infiltration from the surface is expected to be rapidly attenuated with depth, leading to predominantly anoxic conditions (Escudero et al., 2018a; Kieft, 2016), recent studies have reported light-independent oxygen production in anoxic environments (Ruff et al., 2024). If such processes occur in the IPB subsurface, even transient or spatially restricted oxygen availability could introduce additional redox complexity, influencing nitrogen, sulfur, iron and carbon transformations within the underground bioreactor.

### 4.2 Role of the isolates in the synthesis of vitamin B_12_

Vitamin B_12_ or cobalamin is an essential cofactor for many enzymes and is therefore critical for cellular metabolism (Sokolovskaya et al., 2020). In the deep subsurface, it is thought to be particularly important and auxotrophic microorganisms would rely on interactions with cobalamin-producing partners to sustain community functioning (Mateos et al., 2023; Sokolovskaya et al., 2020). Therefore, the genome of the isolates was examined to determine whether any of them can synthesize vitamin B_12_.

It is noteworthy that only *C. telavivensis* sp. T1.2D-1 contains the genes of the anaerobic biosynthetic pathway while *Pleomorphomonas* sp. T1.2MG-36, *P. aeruginosa* sp. T2.1D-1.1 and *R. serenus* sp. P11 contain the genes of the aerobic pathway, which requires the presence of oxygen (Balabanova et al., 2021). However, as previously discussed, light-independent oxygen production has been reported in anoxic environments so the aerobic pathway may still operate at depth (Ruff et al., 2024). These isolates could therefore serve as potential cobalamin producers within the IPB subsurface.

Although only four of the microorganisms seem to be able to synthesize this vitamin *de novo*, all the microorganisms studied encode in their genomes transporters that enable its uptake from the extracellular medium. This suggests that a limited number of producers may support a broader network of cobalamin-dependent taxa. Such cofactor-mediated dependencies further reinforce the view of the IPB subsurface as an interconnected metabolic system in which community stability relies not only on redox coupling, but also on the exchange of essential metabolites.

### 4.3 Genomic similarity

*R. serenus* P11 and *T. lapidicaptus* O5.2 were compared with two strains of the same species, *R. serenus* T2.26MG-98 and *T. lapidicaptus* T2.5–3, to study how similar the isolates of each species were to one another despite being isolated from different depths. The first two microorganisms were isolated from an enrichment culture for microorganisms capable of conducting NDFO, inoculated with samples from borehole BH10 at a depth of 413.3 mbs (Martínez-Bonilla, 2025). Meanwhile, *R. serenus* T2.26MG-98 and *T. lapidicaptus* T2.5–3 were isolated from rock samples collected from depths of 492.6 and 139.4 mbs, respectively (Amils et al., 2023). The genomic indexes showed high values in both cases, indicating that the two isolates from each species are very close taxonomically to each other (Table 3). Despite this high genomic similarity, the isolates represent different strains that differ in their metabolic potential, indicating functional divergence at the strain level. For instance, both isolates of *R. serenus* are capable of denitrification and can reduce tetrathionate. However, *R. serenus* T2.26MG-98 is also capable of using S_2_O_3_^2−^ as electron donor (Amils et al., 2023). In the case of *T. lapidicaptus*, both are capable of NO_3_^−^ reduction to NO_2_^−^, but *T. lapidicaptus* T2.5-30 is also capable of reducing NO_2_^−^ to NH_4_^+^ and NO (Amils et al., 2023).

The occurrence of closely related strains at different depths throughout borehole BH10 indicates that these species are broadly distributed within the IPB subsurface. Nevertheless, the observed strain-level differences in metabolic potential suggest fine-scale functional adaptation, likely reflecting responses to depth-dependent environmental conditions within this ecosystem.

### 4.4 Environmental relevance of the isolates in the underground bioreactor of the IPB

Beyond individual metabolic capabilities and strain-level differences, the vertical distribution and activity patterns of these microorganisms provide insight into the functioning of the IPB underground bioreactor as an integrated system. Each microorganism analyzed in this study was isolated from different depths of the subsurface of the IPB (Amils et al., 2023). Multiple lines of evidence indicate that several of these taxa are not restricted to a single depth but are vertically distributed across the IPB subsurface. During the IPBSL drilling project, a combination of molecular and microbiological techniques was applied to investigate the vertical distribution of microbial genera along borehole BH10 (Amils et al., 2013, 2023).

These analyses revealed that genera such as *Pseudomonas, Rhodoplanes* and *Tessaracoccus* are widely distributed throughout the deep subsurface of the IPB, which was further confirmed by Fluorescence *in situ* Hybridization (FISH) (Figure 2). The broad distribution of these genera suggests that they are well adapted to subsurface conditions. Still, the distribution of the remaining genera analyzed here (*Aestuariimicrobium, Cellulomonas, Lelliottia, Microbacterium, Niallia, Nocardioides, Paenibacillus* and *Propionicimonas*) has not yet been assessed using hybridization-based approaches. While these taxa may also occur across multiple depths, this remains to be experimentally validated. Interestingly, the detection of the same species throughout the borehole does not necessarily indicate functional equivalence. Strains occurring at different depths may differ in their metabolic potential and activity, as demonstrated by the strain-level differences observed for *R. serenus* and *T. lapidicaptus*.

Metabolic activities across different depths were investigated during the IPBSL project by activating microorganisms in rock samples through the addition of mineral medium and by establishing enrichment cultures. These experiments revealed significant denitrification activity at 352.7 and 568.6 mbs, pronounced Fe(II) oxidation at 311.1 mbs, and H_2_ production at 228, 416, and 492 mbs. Additionally, CO_2_ production was detected throughout the borehole, with particularly high concentrations at 90, 266.3, 284 and 352.7 mbs (Figure 2). Notably, several of these depths-specific activities coincide with the presence of the genera analyzed in this study (Figure 2). For example, at depths showing high denitrification activity, genera such as *Pseudomonas* and *Rhodoplanes* were present. Genomic analysis confirmed that isolates from these genera possess the necessary genes for denitrification (Figure 1), suggesting they could be involved in this metabolism at those depths. Given the widespread occurrence of NO_3_^−^ in the subsurface and the presence of multiple nitrate-reducing microorganisms identified in this study, denitrification is likely not restricted to these depths but may occur throughout borehole BH10 and other areas of the IPB subsurface. Likewise, at depths showing Fe(II) oxidation, *Nocardioides*, *Propionicimonas*, *Pseudomonas*, and *Rhodoplanes* were identified, consistent with the potential for NDFO as discussed above and with the elevated Fe(II) concentrations detected in the subsurface. The co-occurrence of Fe(II), NO_3_^−^ and nitrate-reducing microorganisms across the borehole further suggests that this metabolism may operate at multiple depths. At depths with high H_2_ production, *Cellulomonas* and *Lelliottia* were detected, suggesting a role in this metabolism. Low and medium production levels were also observed throughout the borehole. At some depths, such as 228 mbs, high H_2_ concentrations were directly measured during the IPBSL project, supporting a biological origin. Similarly, at depths with high CO_2_ production, genera such as *Cellulomonas*, *Microbacterium*, *Niallia*, *Pseudomonas* and *Tessaracoccus* were also identified, which could contribute to CO_2_ release through fermentative metabolisms or catabolic pathways like the Krebs cycle. Moreover, carbohydrates (sugars) and CO_2_ were detected at significant concentrations in borehole BH10, indicating that CO_2_ production may be linked to microbial utilization of organic substrates via fermentation or anaerobic heterotrophic respiration (Figure 2).

**Figure 3:**
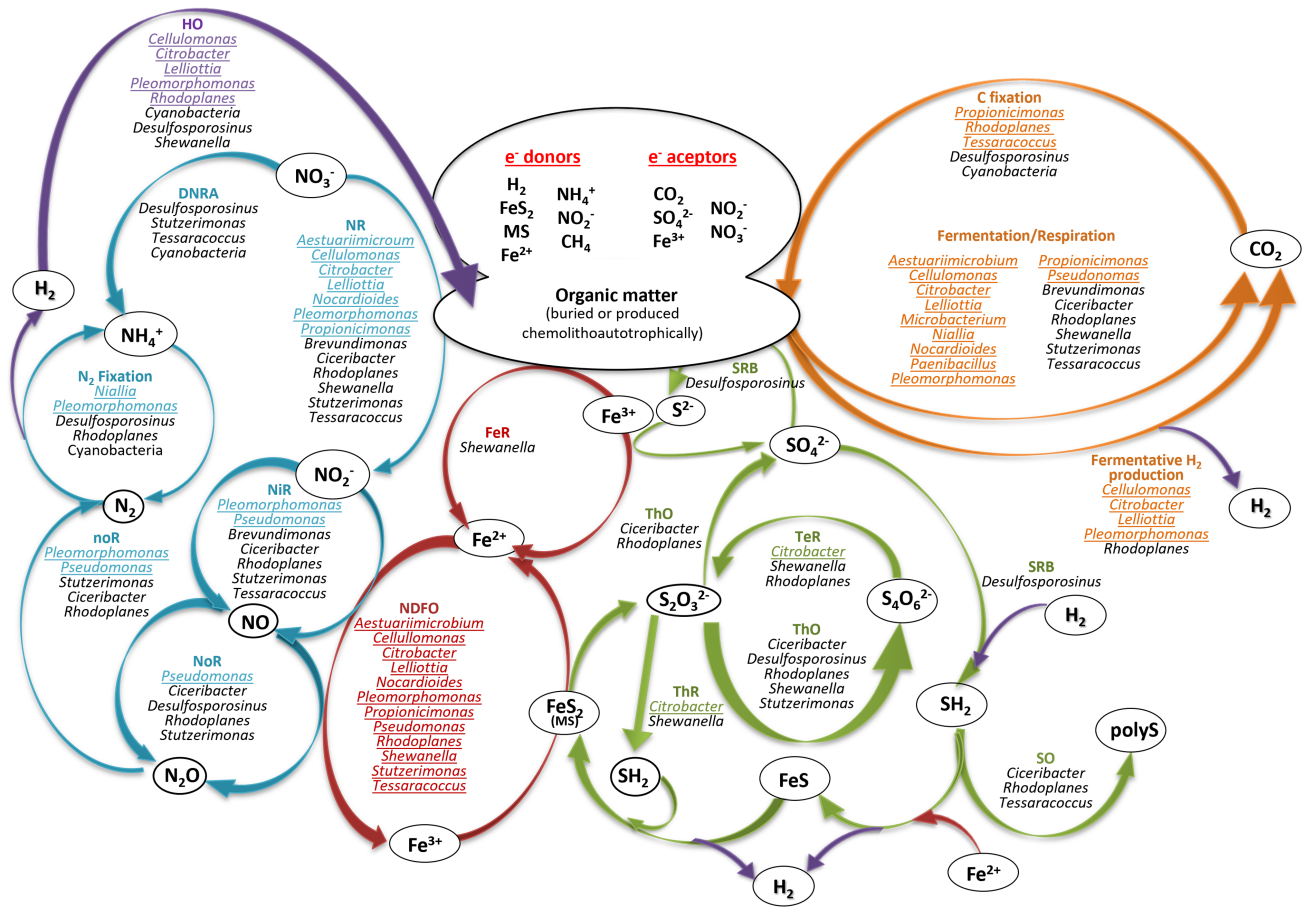
Representation of the metabolic cycles operating in the deep subsurface of the Iberian Pyrite Belt. The figure includes the metabolic processes that the 22 microorganisms sequenced and analyzed so far from the IPB deep subsurface may potentially perform in this ecosystem. The displayed metabolisms are associated with the nitrogen (blue), iron (red), sulfur (green), carbon (orange), and hydrogen (purple) cycles. Only genus names are shown. Underlined and bold names correspond to the isolates analyzed in this study, whereas the remaining genera correspond to previously published microorganisms. The following abbreviations are used: HO, hydrogen oxidation; DNRA, dissimilatory nitrate reduction to ammonium; NR, nitrate reduction; NiR, nitrite reduction; NoR, nitric oxide reduction; noR, nitrous oxide reduction; FeR, Fe reduction; NDFO, nitrate dependent Fe(II) oxidation; SRB, sulfate reducing bacteria; SO, sulfur oxidation; TeR, tetrathionate reduction; ThO, thiosulfate oxidation; ThR, thiosulfate reductase; and MS, metallic sulfides. Modified from Amils et al. (2023) and updated with the genomic information generated in this study.

By integrating the metabolic potential inferred from the genomes analyzed here with previously published genomic data from the IPB deep subsurface, it is possible to reconstruct a network of potential metabolic interactions operating within this ecosystem (Figure 3). This conceptual model illustrates how different biogeochemical cycles could be interconnected, allowing microbial communities to thrive in the absence of direct nutrients inputs from the surface. Under these conditions, metabolic cooperation, cross-feeding and the recycling of metabolic intermediates become essential for maintaining the ecosystem function.

The results of this work further support the relevance of the nitrogen cycle in the deep subsurface of the IPB, as reflected on the number of isolates that can potentially take part in nitrogen transformations. Different nitrogen species likely act as key electron donors and acceptors in this environment and may also link nitrogen cycling to other processes, such as NDFO and anaerobic pyrite oxidation, which have been proposed as drivers of the extreme geochemical conditions observed in the Río Tinto system.

Although rare cases of metabolically autonomous microorganisms have been reported in the deep subsurface (Chivian et al., 2008), such autonomy is unlikely to be widespread in the IPB. Instead, different microorganisms in this system co-habit microniches within the rock matrix, often forming biofilms (Escudero et al., 2018b). These biofilms create localized microenvironments that can facilitate metabolic interactions, stabilize redox gradients, and support diverse physiological strategies. Thus, biofilms-based life likely play an important role in the deep subsurface of the IPB, as well as in other deep subsurface environments (Casar et al., 2021).

## 5 Conclusion

In the deep subsurface of the IPB, there is an active underground bioreactor, which is the primary driver of the extreme physicochemical conditions observed in Río Tinto. The results presented here provide additional genomic and ecological evidence supporting this concept. Five microorganisms isolated from this environment were taxonomically classified at the species level. In addition, seven additional isolates have been identified as putative novel species, although further characterization will be required to confirm this result.

The detection of certain genera and species at multiple depths suggests a broad ecological distribution, although strain-level differences in metabolic potential indicate functional differentiation rather than uniform activity throughout the subsurface.

Among the studied isolates, several represent potential key contributors to the C (carbon fixation via the Calvin cycle), H (hydrogen production), N (denitrification and nitrogen fixation), S (thiosulfate and tetrathionate reduction) and Fe (Nitrate Dependent Fe(II) oxidation) cycles. In addition, some isolates might also contribute to vitamin B_12_ production, further supporting the existence of metabolic interdependencies within the subsurface community.

Overall, by sustaining interconnected biogeochemical cycles and metabolic interactions within the underground bioreactor, the microorganisms studied here are likely to play a significant role in shaping the geochemical conditions of the IPB subsurface and, ultimately, in the origin of the extreme characteristics observed in Río Tinto.

## Data Availability

The datasets presented in this study can be found in online repositories. The names of the repository/repositories and accession number(s) can be found below: https://www.ncbi.nlm.nih.gov/genbank/, GCA_950101875.1 https://www.ncbi.nlm.nih.gov/genbank/, GCA_964266755.1 https://www.ncbi.nlm.nih.gov/genbank/, GCA_950101345.1 https://www.ncbi.nlm.nih.gov/genbank/, GCA_950097975.1 https://www.ncbi.nlm.nih.gov/genbank/, GCA_950097965.1 https://www.ncbi.nlm.nih.gov/genbank/, GCA_950101795.1 https://www.ncbi.nlm.nih.gov/genbank/, MF361877.1 https://www.ncbi.nlm.nih.gov/genbank/, GCA_950100655.1 https://www.ncbi.nlm.nih.gov/genbank/, GCA_964266745.1 https://www.ncbi.nlm.nih.gov/genbank/, GCA_950101725.1 https://www.ncbi.nlm.nih.gov/genbank/, GCA_964266735.1 https://www.ncbi.nlm.nih.gov/genbank/, GCA_964266725.1.
