# Supplementary material for: Metabolic potential of newly isolated bacterial species from the deep subsurface of the Iberian Pyrite Belt
Source: Front Microbiol. 2026 Jun 9;17:1822116. doi: 10.3389/fmicb.2026.1822116 (PMC13286885; doi:10.3389/fmicb.2026.1822116)

**Metabolic potential of newly isolated bacterial species from the deep subsurface of the Iberian Pyrite Belt**

Supplementary Material


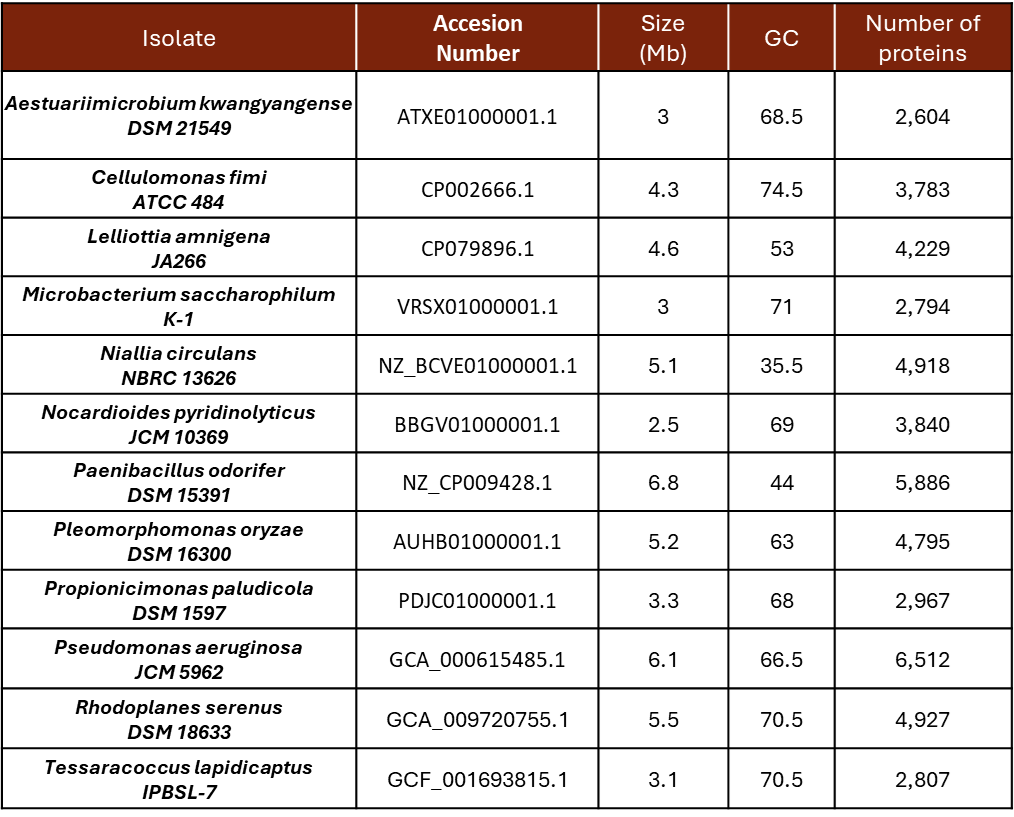


Supplementary Table S1. Isolates used as reference to calculate the genomic indexes for the microorganisms studied in this project. The table shows the accession number from GenBank for each of the genomes, the length of the assembled genome in Mb, the content of GC (%) and the number of proteins coded in the genome.

**
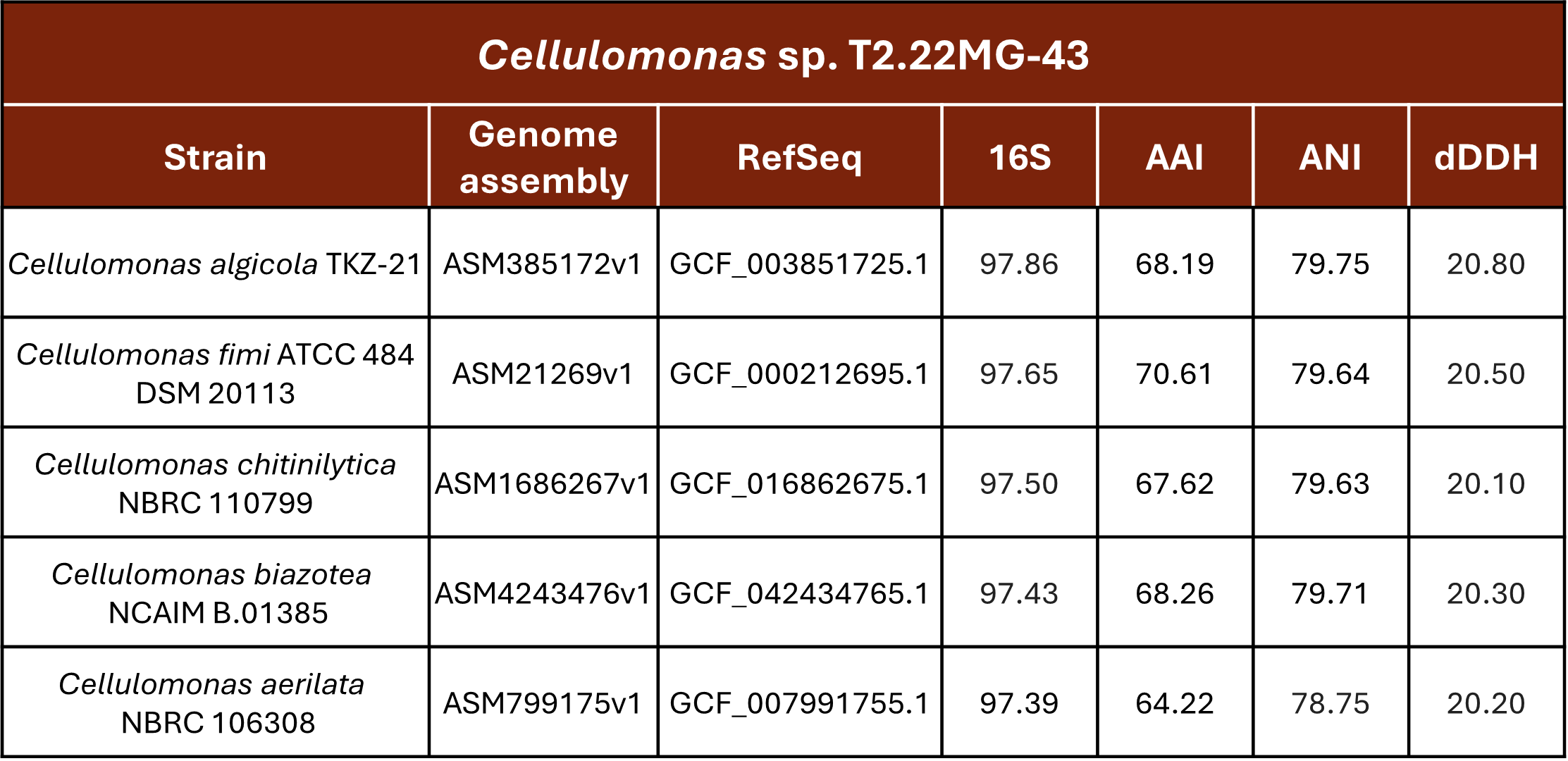

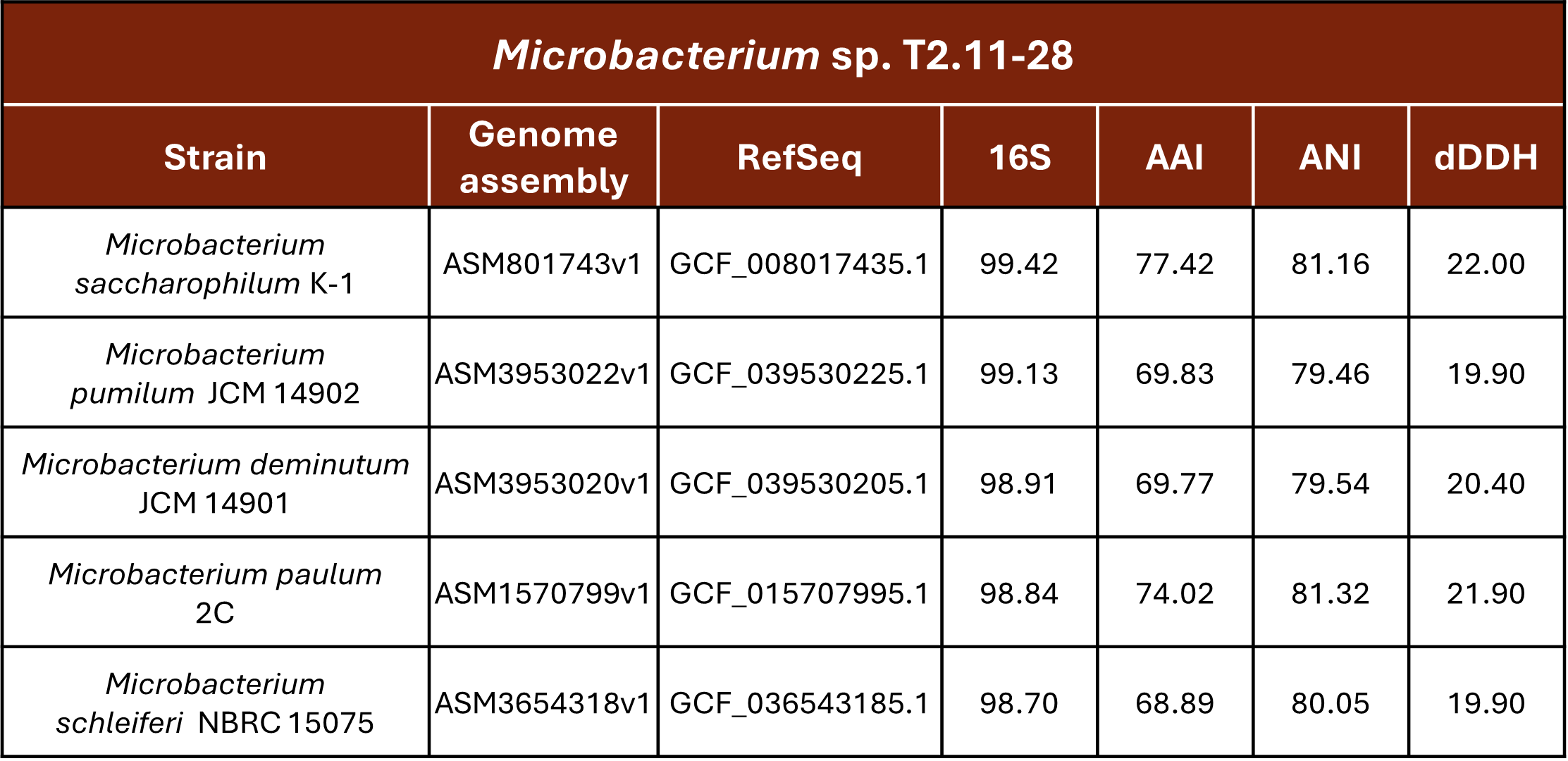
**

**
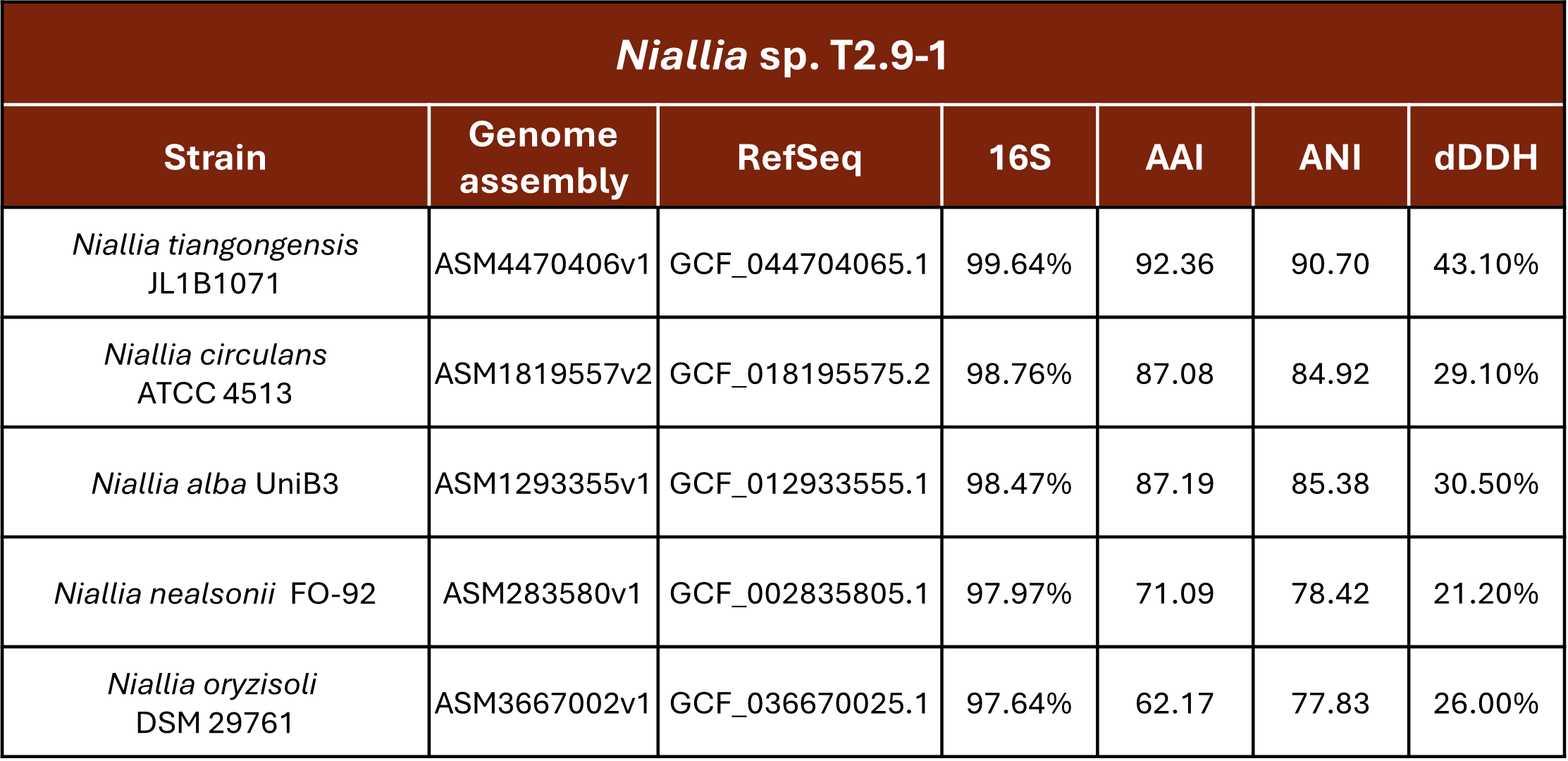
**

**
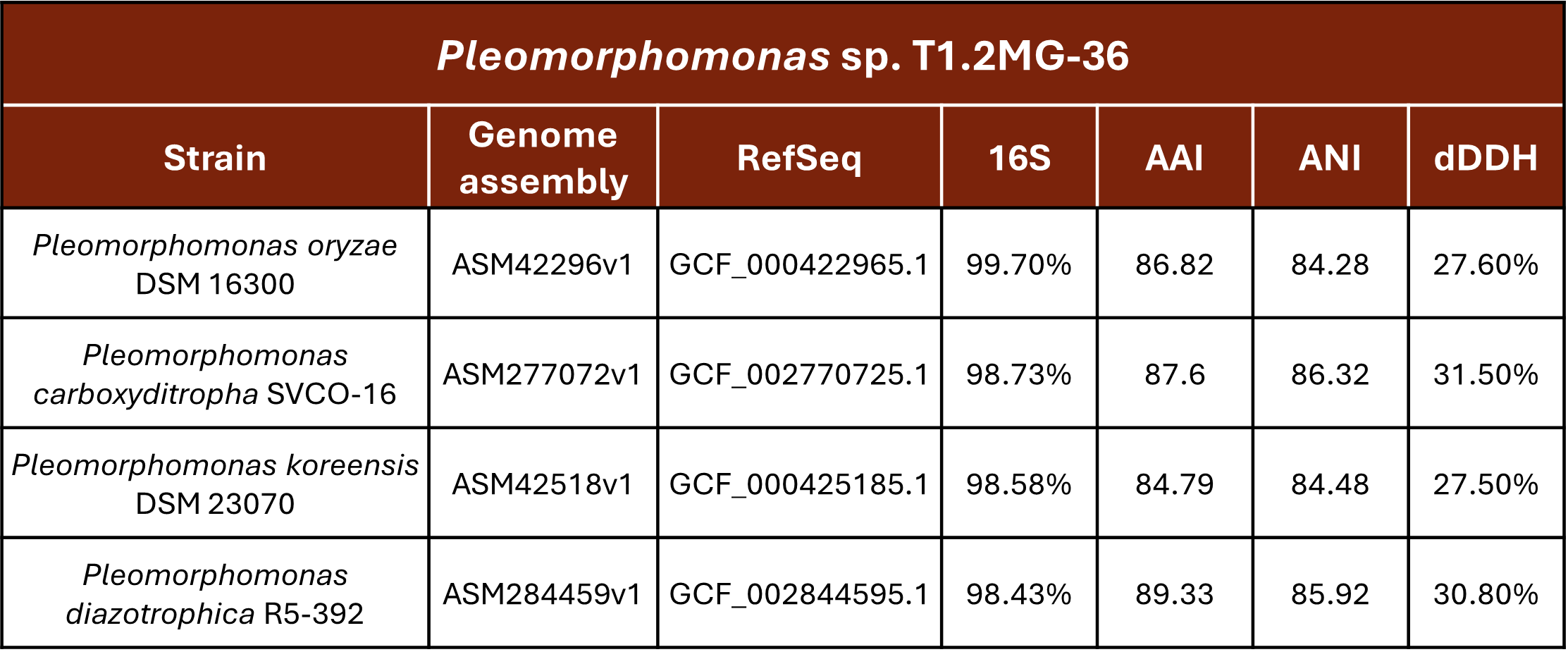

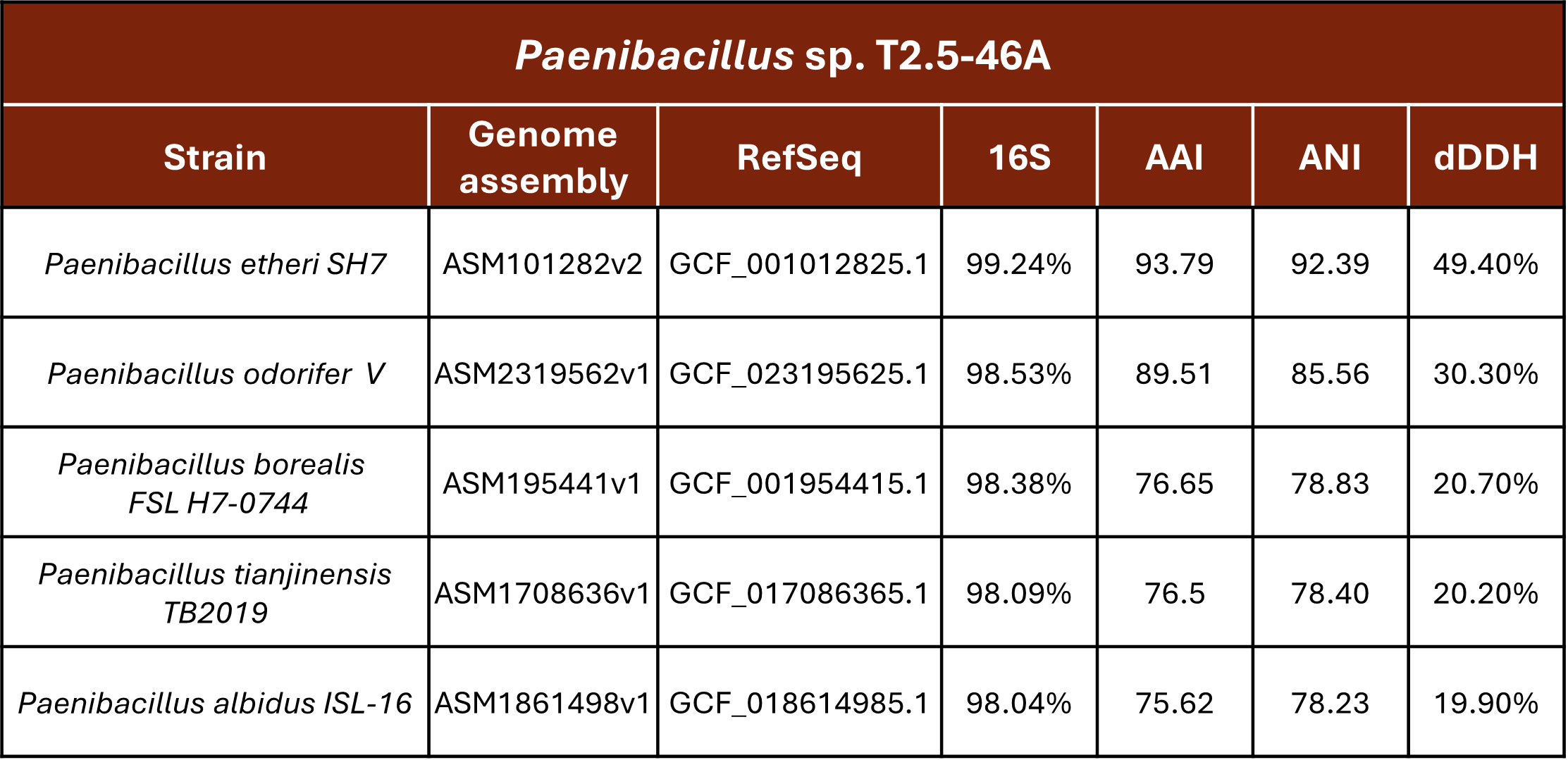

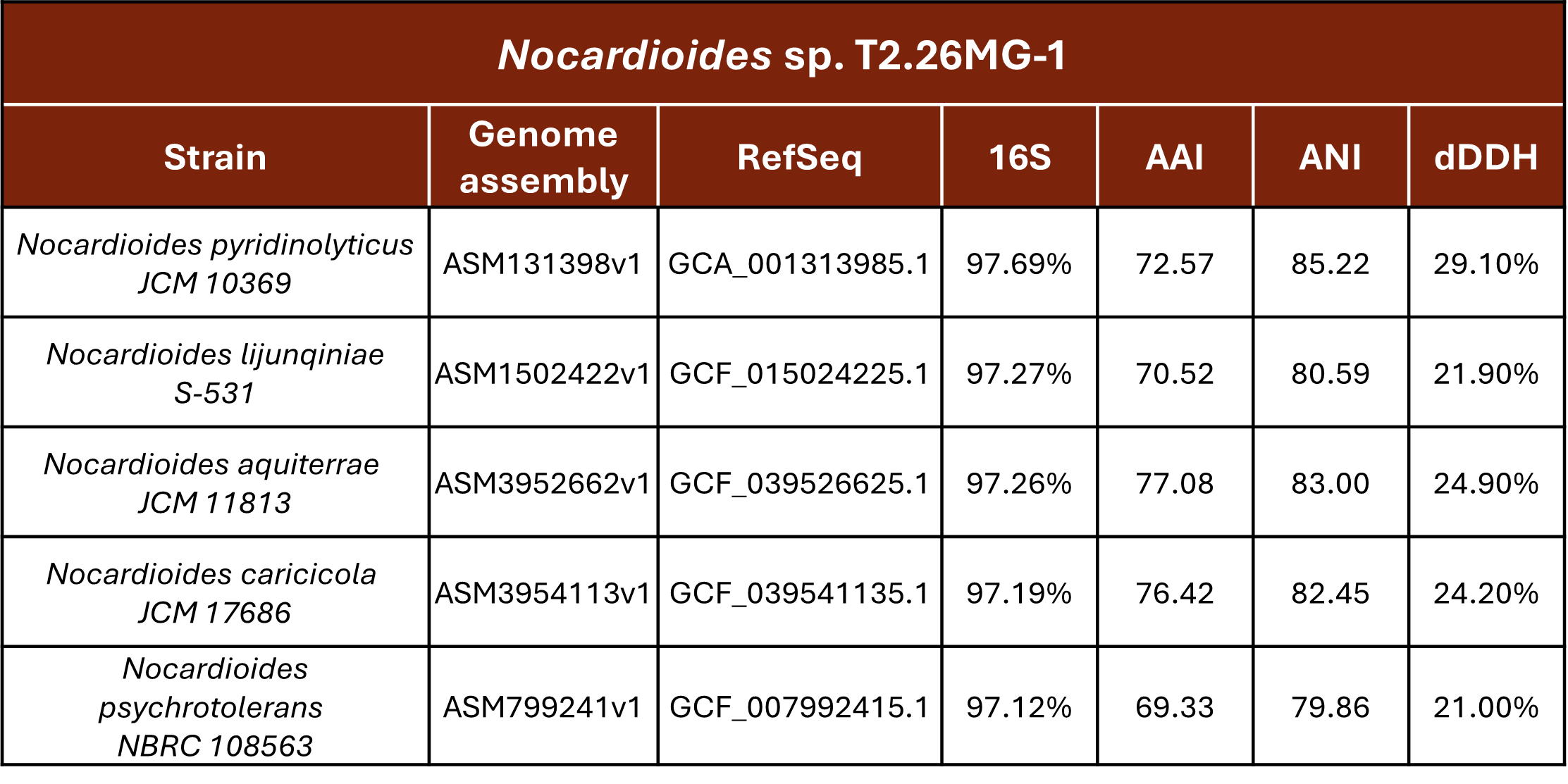
**

Supplementary Table S2. Additional genomic indexes calculated for the unclassified isolates. The tables show the results of the comparison of the six isolates that could not be taxonomically classified with the five closest species based on their 16S rRNA gene. The tables show the percentage of sequence identity of the 16S rRNA gene and the corresponding AAI, ANI and dDDH values.

**Supplementary Figure 1. Metabolic potential of the microorganisms isolated from the deep subsurface of the IPB.** The figure shows the genes searched for in the genomes of the microorganisms studied in this project. In particular, genes from the nitrogen (blue), sulfur (green), hydrogen (purple), iron (red) and carbon (orange) cycles. Also genes related to the synthesis and uptake of vitamin B_12_ (grey). In the case of vitamin B_12_, the core set of genes found in synthesizers (Shelton et al., 2019) are marked in bold. A number was assigned to each of the isolates, as shown in the first part of the figure. The tables show the different studied genes, the pathway they are related to and the enzyme encoded by those genes. Coloured circles mark the presence of the gene in the genome of the isolate.

1. *Aestuariimicrobium kwangyangense* T2.26MG-19.2B
2. *Cellulomonas* sp. T2.22MG-43
3. *Citrobacter telavivensis* T1.2D-1
4. *Lelliottia amnigena* T2.23D-8
5. *Microbacterium* sp. T2.11-28
6. *Niallia* sp. T2.9-1
7. *Nocardioides* sp. T2.26MG-1
8. *Paenibacillus* sp. T2.5-46A
9. *Pleomorphomonas* sp. T1.2MG-36
10. *Propionicimonas* sp. T2.31MG-1
11. *Pseudomonas aeruginosa* T2.1D-1.1
12. *Rhodoplanes serenus* P11
13. *Tessaracoccus lapidicaptus* O5.2


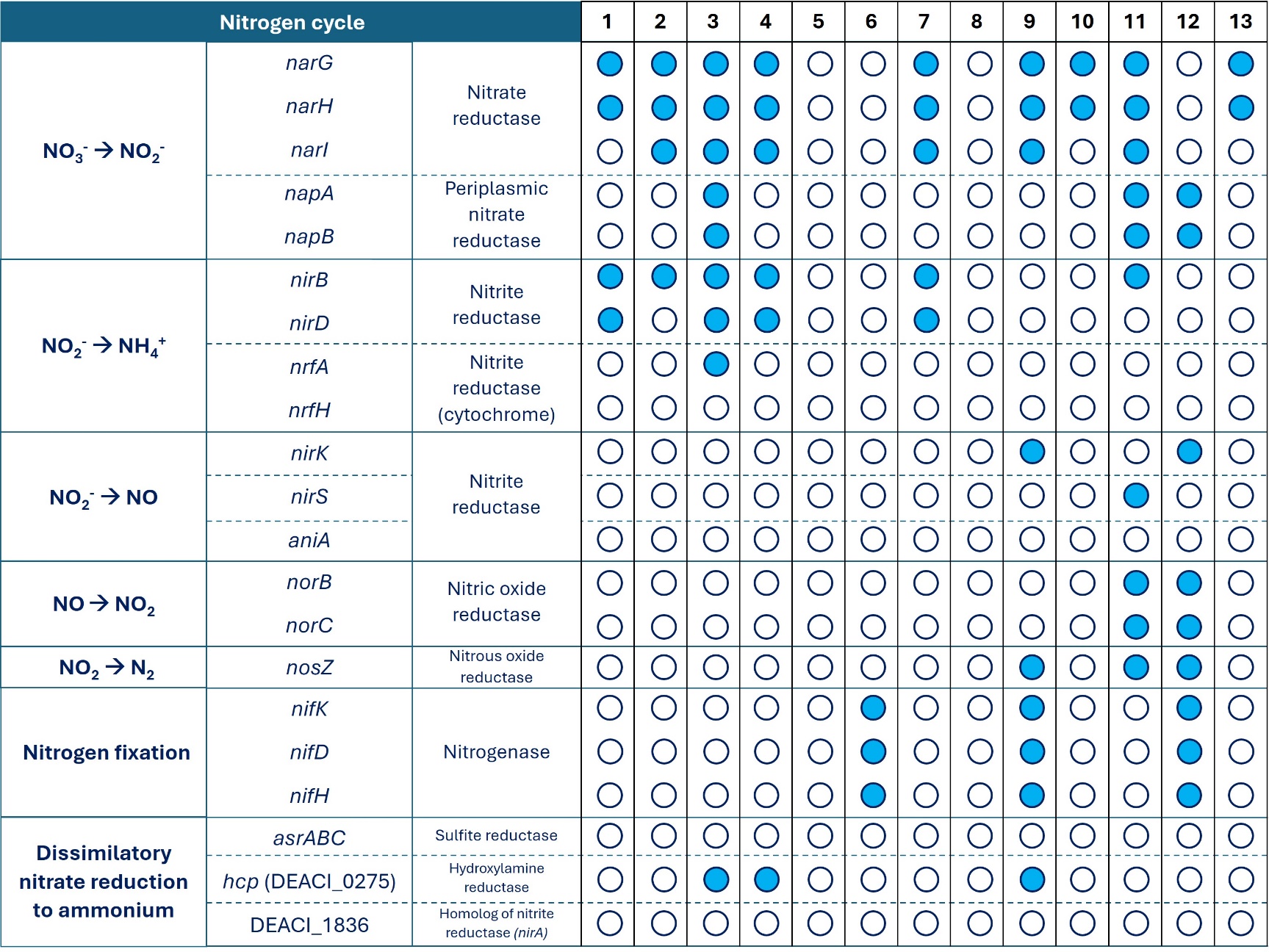


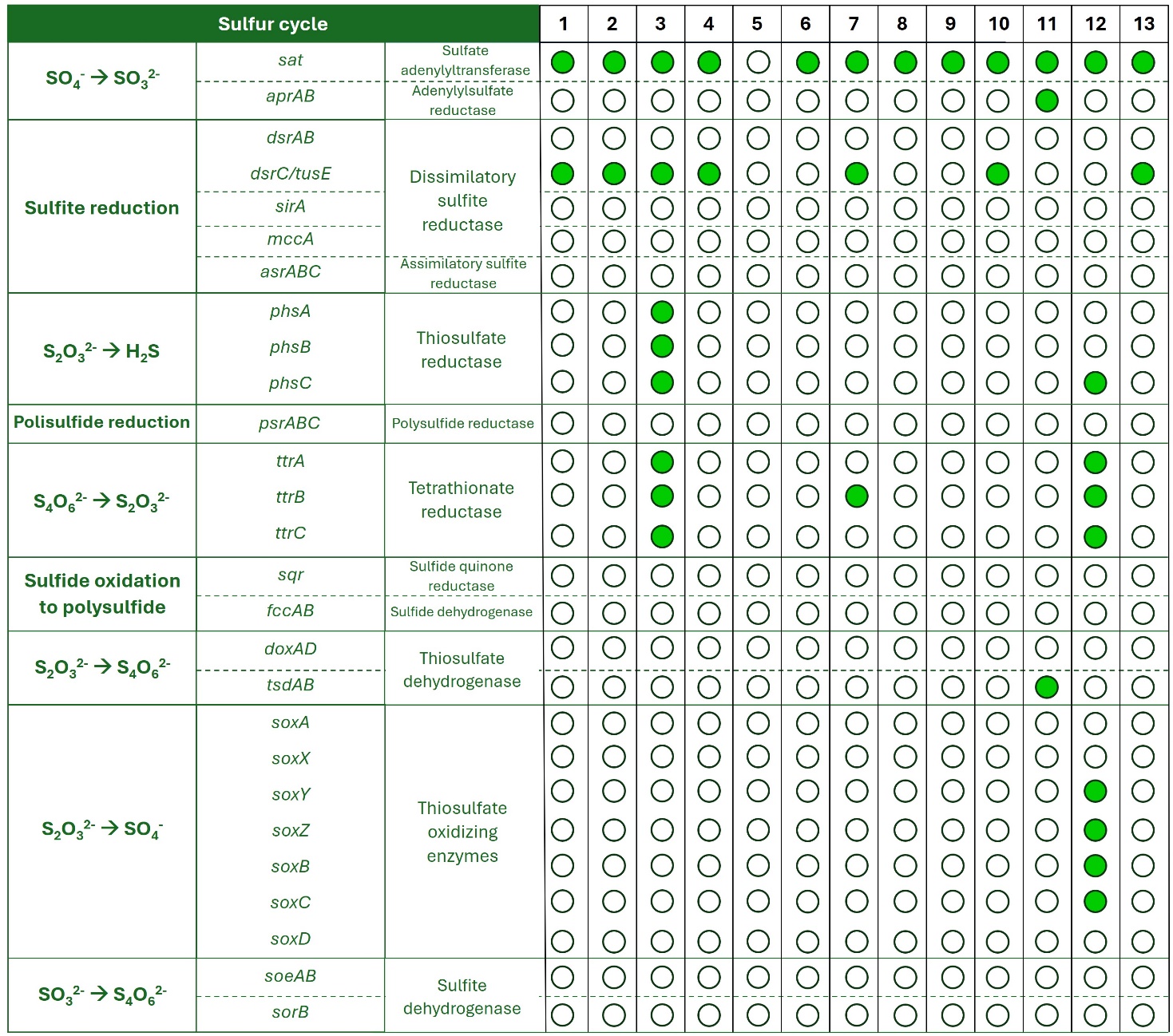


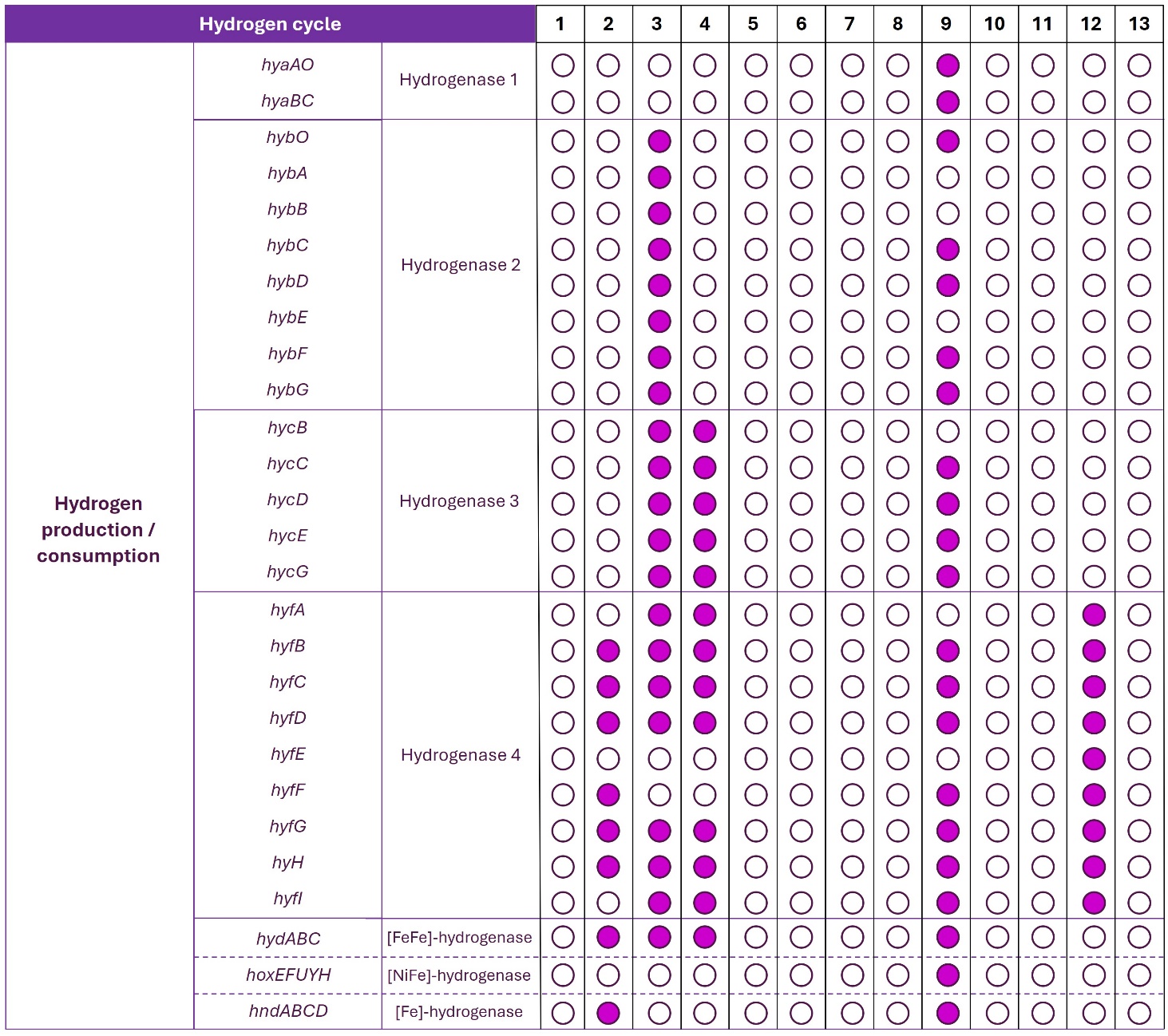


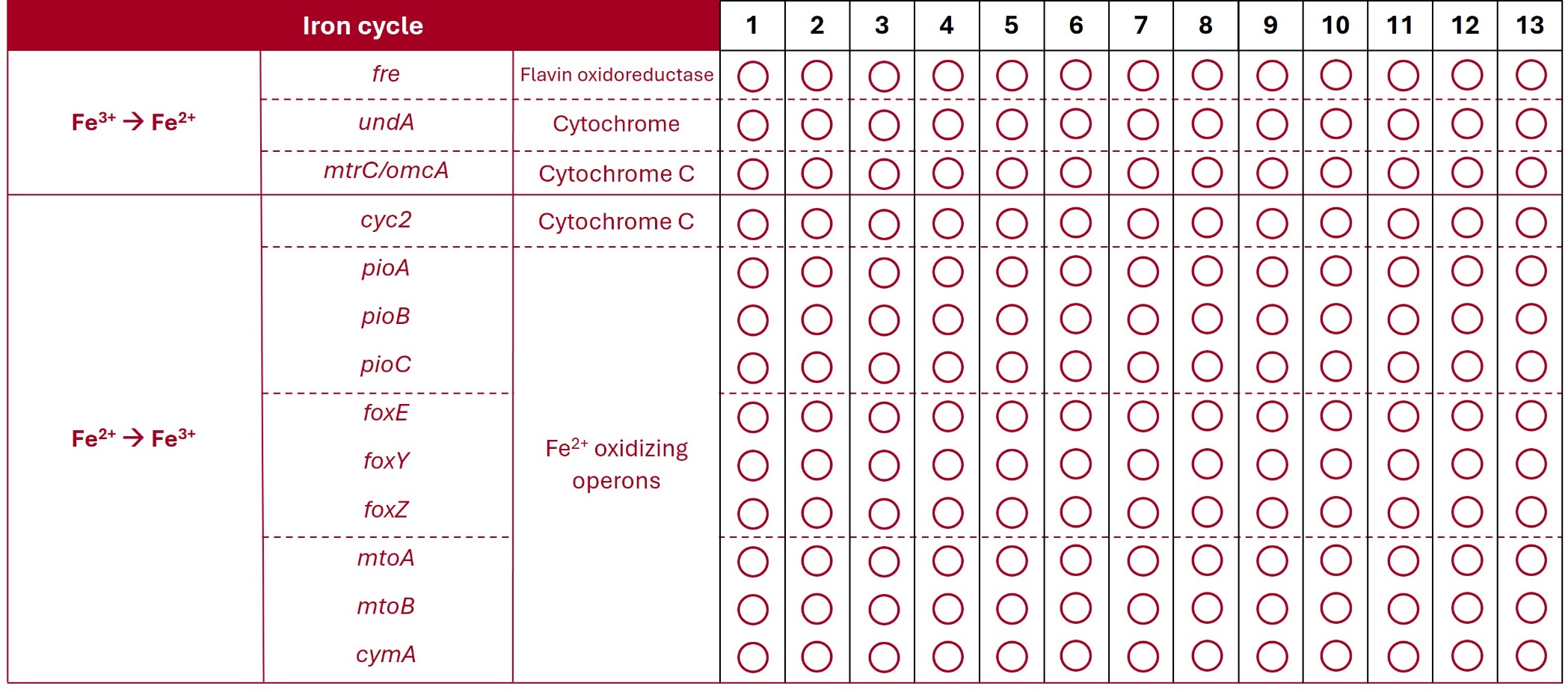


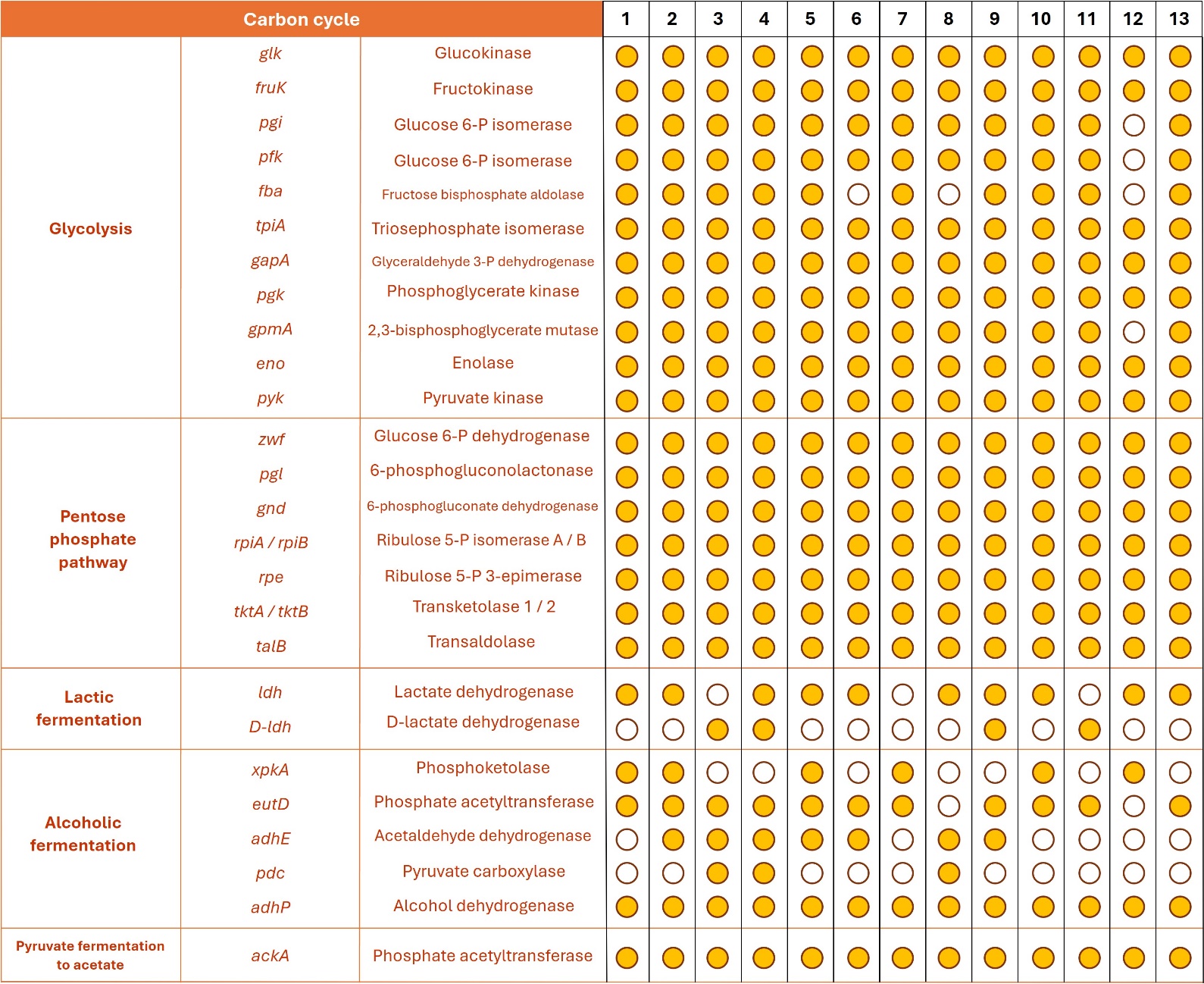


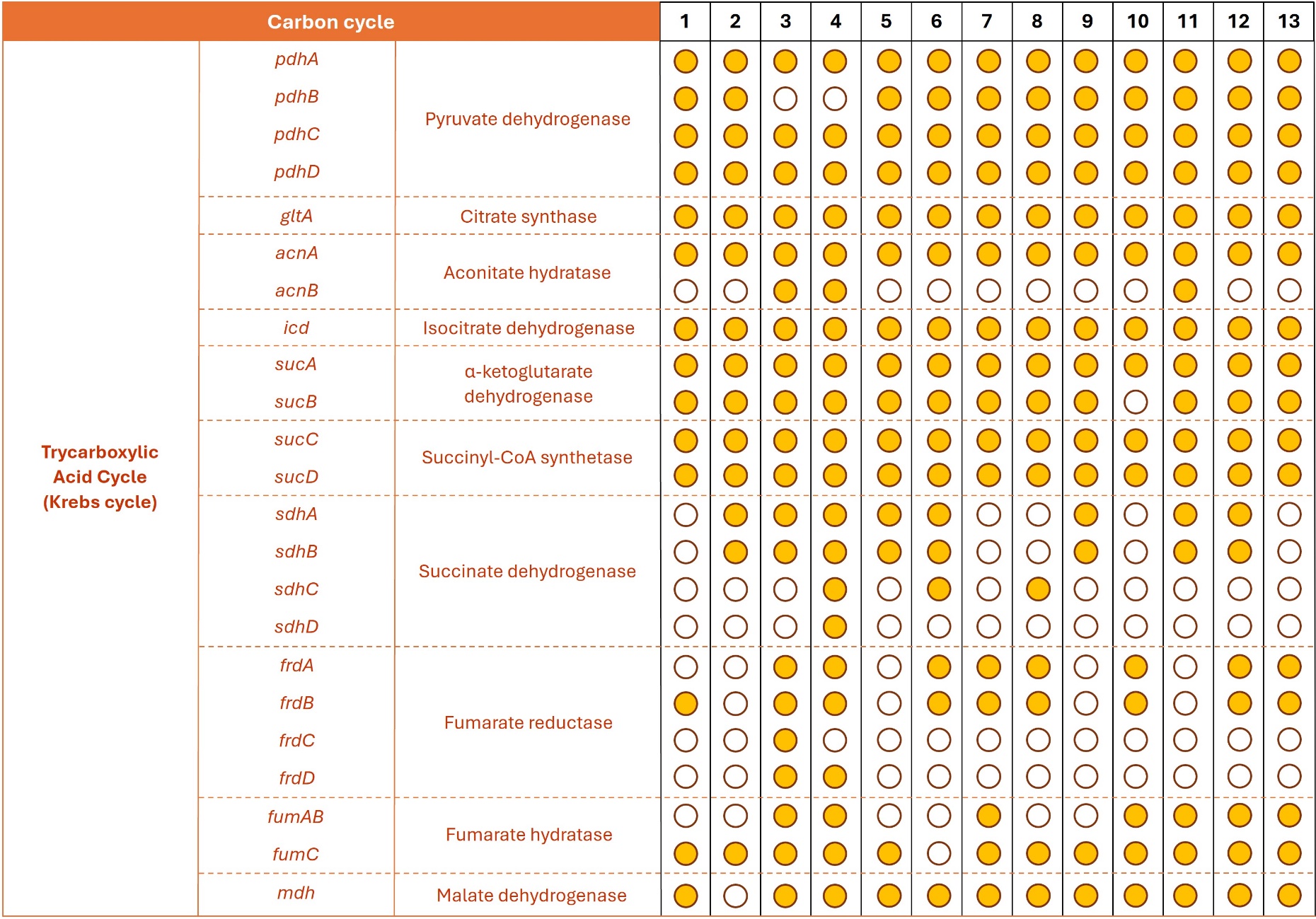


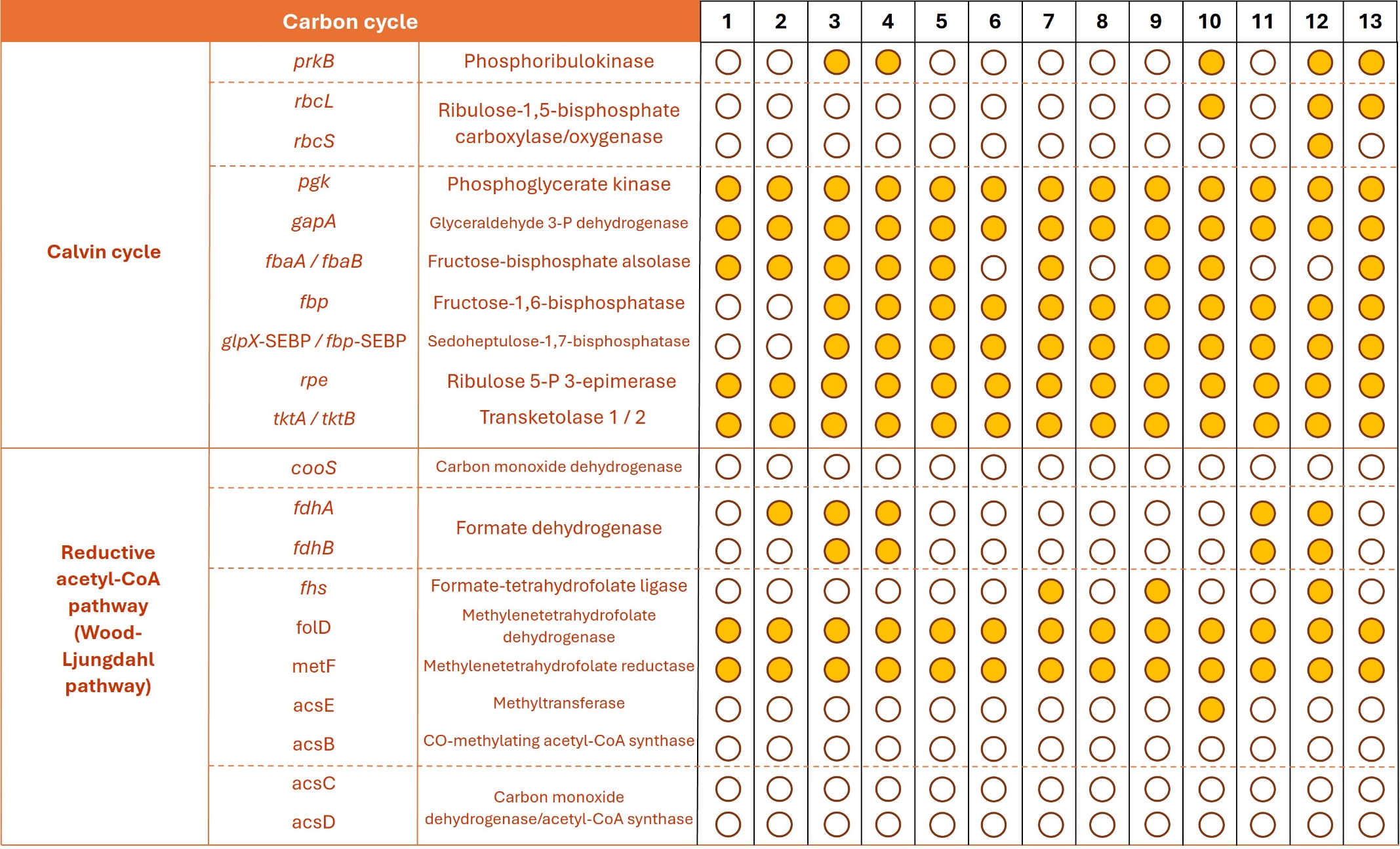


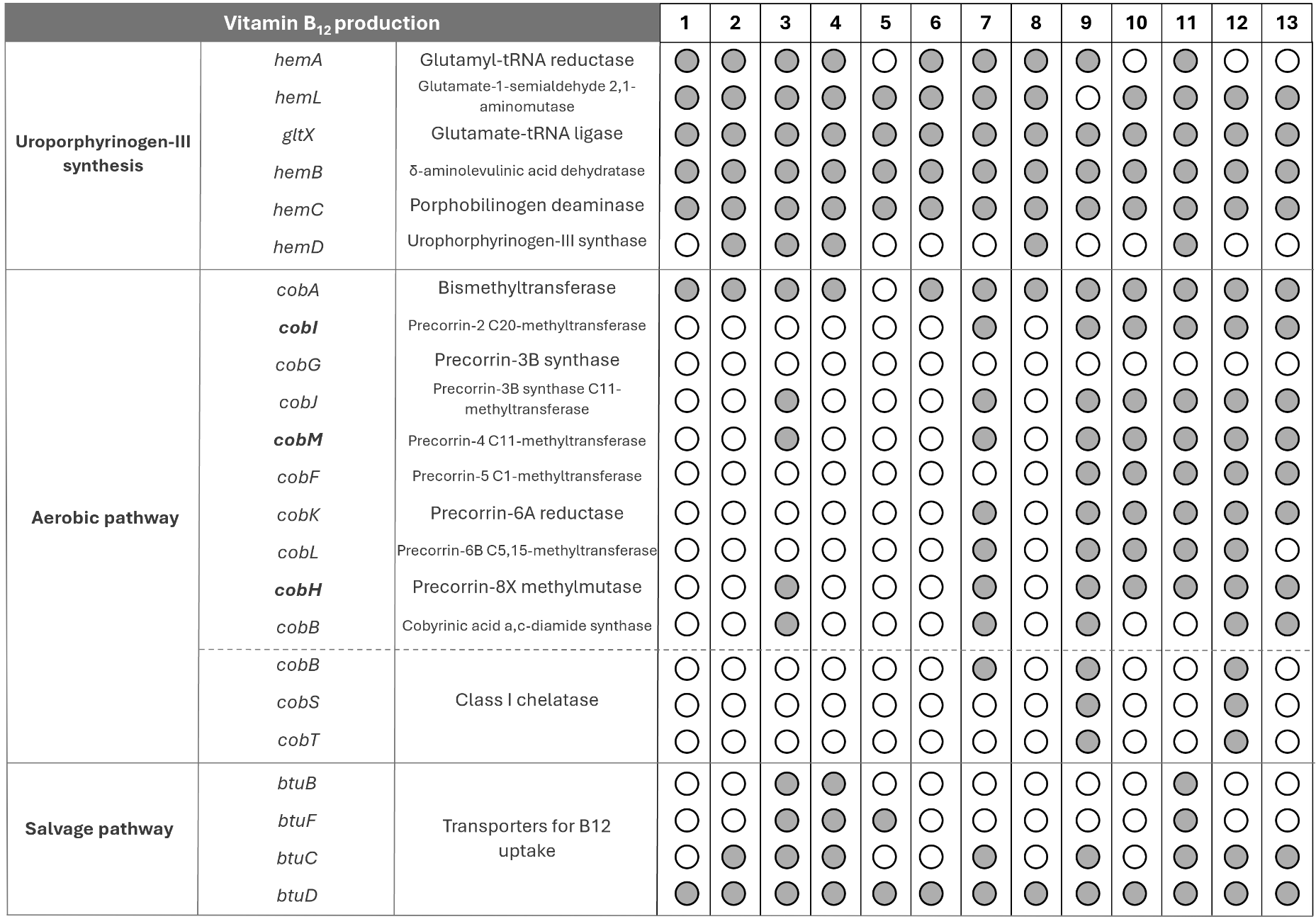


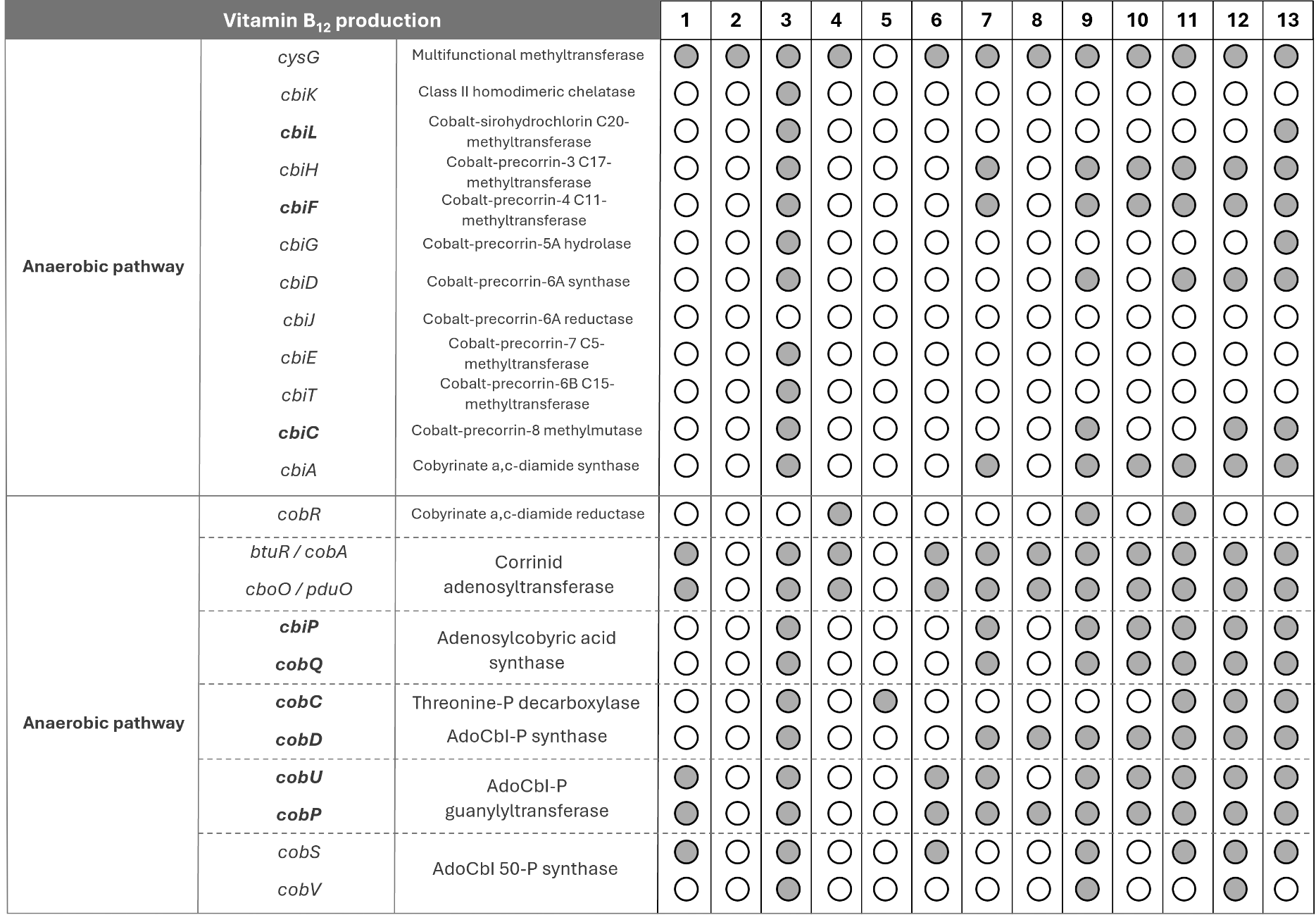

Supplement: Supplementary file 1 [file Data_Sheet_1.docx]
